# Supplementary material for: Evolution of the patellar sesamoid bone in mammals
Source: PeerJ. 2017 Mar 21;5:e3103. doi: 10.7717/peerj.3103 (PMC5363259; doi:10.7717/peerj.3103)
Supplement: Supplemental Information 2 — Supplementary Tables S1 and S2; Supplementary Figures S1–S5; and Supplementary References. [file peerj-05-3103-s002.pdf]

Evolution of the patellar sesamoid bone in mammals

SUPPLEMENTARY INFORMATION: Tables, Figures and References

Supplementary Table S1: Mammaliaform patellar status[$]

Inclusive clades (partial)	Genus and species (and reference(s) used for patellar status)	Stratigraphic age of taxon, and location(s)	Patellar state 0/1/2 (absent/ 'patelloid'/ present)	Comments[#]
Sinoconodonta	*Sinoconodon rigneyi* (Kielan-Jaworowska et al., 2004)	Jurassic, China	0	Patellar groove absent, suggests no patella<br>*Sinoconodon* is included on our phylogeny within tritylodontids.
Morganucodonta	*Megazostrodon rudnerae* (Jenkins & Parrington, 1976)	Late Triassic, southern Africa	0	
Morganucodonta	*Eozostrodon sp.* (Jenkins et al., 1976)	Late Triassic, Wales	0	Asymmetric patellar groove, specimens disarticulated so it is hard to assess the patella but appears absent
Docodonta	*Castorocauda lutrasimilis* (Ji et al., 2006)	164 Mya, mid-Jurassic, China	0	Semi-aquatic adaptations
Docodonta	*Agilodocodon scansorius* (Meng et al., 2015)	164 Mya, mid-Jurassic, China	0	
Docodonta	*Docofossor brachydactylus* (Luo et al., 2015b)	160 Mya, China	0	
Docodonta	*Haldanodon exspectatus* (Martin, 2005b)	150-155 Mya, Late Jurassic, Portugal	0	Shallow patellar groove
Australosphenida	*Asfaltomylos patagonicus* (Martin, 2005a)	Mid-Jurassic, South America	?	Postcranial material absent
Australosphenida Monotremata	*Ornithorhynchus anatinus* (Herzmark, 1938; Rowe, 1988)	Extant	2	Platypus, genome sequenced (Warren, Hillier, Marshall Graves *et al.*, 2008)
Australosphenida Monotremata	*Tachyglossus* + *Zaglossus* spp. (Herzmark, 1938;	Extant	2	Echidnas

	Rowe, 1988)			
Mammaliaformes indet.	*Fruitafossor windscheffeli* (Luo & Wible, 2005)	150 Mya, Late Jurassic, Colorado	0	Phylogenetic position uncertain
Mammaliaformes indet.	*Volaticotherium antiquus* (Meng et al., 2006)	Late Jurassic/Early Cretaceous	?	Hindlimb material incomplete
Eutriconodonta	*Jeholodens jenkinsi* (Ji et al., 1999)	120-125 Mya, Early Cretaceous, China	0	Poorly developed patellar groove
Eutriconodonta	*Gobiconodon* spp. (Jenkins & Schaff, 1988)	100-110 Mya, Early Cretaceous, Montana	0	Shallow patellar groove
Eutriconodonta	*Liaoconodon hui* (Meng et al., 2011)	120-125 Mya, Early Cretaceous, China	0	No patella visible in figure of well-preserved hindlimb
Eutriconodonta	*Yanoconodon allini* (Luo et al., 2007)	120-125 Mya, Early Cretaceous, China	0	
Eutriconodonta	*Repenomamus giganticus* (Hu et al., 2005)	Early Cretaceous, China	0	
Allotheria "Haramiyida"	*Haramiyavia* (Butler, 2000; Jenkins et al., 1997)	Late Triassic, Greenland	?	
Allotheria "Haramiyida" (?)	*Megaconus mammaliaformis* (Zhou et al., 2013)	Mid-Jurassic, China	0	No patella visible in figure of well-preserved hindlimb
Allotheria Euharamiyida	*Arboroharamiya jenkinsi* (Bi et al., 2014; Zheng et al., 2013)	Mid-late Jurassic, China	0	No patella visible in figure of well-preserved hindlimb
Allotheria Euharamiyida	*Shenshou lui* (Bi et al., 2014)	Mid-Jurassic, China	0	Coded as absent by Bi et al. (2014); no patella visible in figure of well-preserved hindlimb
Allotheria Euharamiyida	*Xianshou* spp. (Bi et al., 2014)	Mid-Jurassic, China	0	Coded as absent by Bi et al. (2014); no patella visible in figure of well-preserved hindlimb
Allotheria Multituberculata	*Rugosodon eurasiaticus* (Yuan et al., 2013)	Late Jurassic, China	0	
Allotheria	*Chulsanbaatar* vulgaris (Kielan-	Late Cretaceous, Mongolia	2	

Multituberculata	Jaworowska & Gambaryan, 1994)			
Allotheria Multituberculata	*Kryptobaatar dashzevegi* (Kielan-Jaworowska et al., 1994)	Late Cretaceous, Mongolia	?	
Allotheria Multituberculata	*Nemegbaatar gobiensis*	Late Cretaceous, Mongolia	?	
Allotheria Multituberculata	*Plagiaulacidans* spp. (Luo et al., 2011)	Mid-Jurassic to Early Cretaceous, China	?	As stated in character matrix of Luo et al. (2011), but stated as absent by others (Yuan et al., 2013)
Allotheria Multituberculata	*Cimolodontans* spp. (Luo et al., 2011)	Cretaceous to Early Cenozoic, China	?	As stated in character matrix of Luo et al. (2011), but stated as absent by others (Yuan et al., 2013)
Allotheria Multituberculata	*Ptilodus kummae* (Jenkins & Krause, 1983)	Late Paleocene, Canada	2	
Allotheria Multituberculata	*Eucosmodon/Stygimys* (Granger & Simpson, 1929; Simpson & Elftman, 1928)	Paleocene, North America	?	Femur has patellar groove, status of patella not reported
Symmetrodonta Spalacotheroidea	*Zhangheotherium quinquecuspidens* (Hu et al., 1997; Luo & Ji, 2005)	120-125 Mya, Early Cretaceous, China	2	Noted by Luo & Ji (2005), not by Hu et al. (1997)
Symmetrodonta Spalacotheroidea	*Maotherium asiaticus* (Ji et al., 2009)	120-125 Mya, Early Cretaceous, China	?	
Symmetrodonta Spalacotheroidea	*Akidolestes cifelli* (Chen & Luo, 2012)	120-125 Mya, Early Cretaceous, China	0	
Eupantotheria	*Henkelotherium guimarotae* (Molinero, 2003; Vazquez-Molinero et al., 2001)	Late Jurassic, Portugal	0	
Eupantotheria	*Vincelestes neuquenianus* (Bonaparte, 2008; Rougier, 1993)	Early Cretaceous, South America	0	
Eupantotheria	Dryolestids (spp).	Late Jurassic to early Cenozoic	?	Hindlimb material missing
“Theria”	*Juramaia sinensis*	160-165 Mya, late	?	Hindlimbs missing, placement

	(Luo et al., 2011)	Jurassic, China		relative to basal Theria uncertain
"Theria"	*Eomaia scansoria* (Ji et al., 2002; O'Leary et al., 2013)	120-125 Mya, Early Cretaceous, China	2	Originally placed in Placentalia, restated as "Theriimorpha" immediately adjacent to basal Theria
Metatheria	*Sinodelphys szalayi* (Luo et al., 2003)	120-125 Mya, Early Cretaceous, China	0	Earliest putative metatherian
Metatheria	*Asiatherium reshetovi* (Szalay & Trofimov, 1996; Trofimov & Szalay, 1994)	80 Mya, late Cretaceous, Mongolia	0	Lacks patellar groove on distal femur
Metatheria Deltatheroidea	*Deltatherium* spp. (Rougier et al., 1998)	Cretaceous, Mongolia	?	
Metatheria	*Pucadelphys andinus* (Macrini, et al., 2007)	Early Paleocene, South America	0	As per character matrices (Luo et al., 2011; Wible et al., 2007)
Metatheria	*Mayulestes ferox* (De Muizon, 1994; De Muizon, 1998)	Early Paleocene, South America	0	Carnivorous marsupial<br><br>As per character matrices (Luo et al., 2011; Wible et al., 2007)
Metatheria	*Herpetotherium fugax* (Horovitz et al., 2008; Horovitz et al., 2009)	Oligocene, Wyoming	0	
Metatheria Sparassodonta	*Callistoe vincei* (Argot & Babot, 2011)	Eocene, Argentina	2	Carnivorous marsupial
Metatheria Sparassodonta	*Prothylacinus patagonicus* (Argot, 2003b)	Early Miocene, Patagonia	2	Carnivorous marsupial
Metatheria Sparassodonta	*Boryhyaena tuberata* (Argot, 2003b)	Early Miocene, Patagonia	?	Carnivorous marsupial
Metatheria Sparassodonta	*Artiodictis sinclairi* (Forasiepi, 2004)	Early Miocene, Argentina	2	Carnivorous marsupial
Metatheria Sparassodonta	*Sipalocyon* sp. (Sinclair, 1905)	Mid-Miocene, South America	2	Originally called "*Amphiproviverra*" (Argot, 2003c)
Metatheria Sparassodonta	*Lycopsis longirostris* (Argot, 2004)	Mid-Miocene, South America	?	Carnivorous marsupial
Metatheria Marsupialia	*Thylacinus cynocephalus* (De	20th century, Australia	0	Thylacine- "Tasmanian tiger" or "wolf"; recently extinct. Patella

Thylacinidae	Vriese, 1909)			fibrocartilaginous? See Fig. S3 Y,Z
Metatheria Marsupialia Didelphidae	*Didelphis virginiana* (Szalay & Sargis, 2001)	Extant, North America	0	North American opossum. Coding for this taxon is debatable—some studies claim it has an ossified patella in older adults (Szalay et al., 2001). More study is needed to confidently code it
Metatheria Marsupialia Didelphidae	*Monodelphis domestica*	Extant, South America	0	Gray short-tailed opossum. Genome sequenced (Mikkelsen, Wakefield, Aken *et al.*, 2007)
Metatheria Marsupialia Didelphidae	*Metachirus nudicaudatus* (Szalay et al., 2001)	Extant, Central America	0	Brown four eyed opossum
Metatheria Marsupialia Macropodidae	*Macropus spp.* and relatives (Reese et al., 2001)	Extant, Australia	1	Kangaroos, wallabies.<br>As noted above for other marsupials and in the main text, coding for this taxon is complicated by the possibility that the patella may only ossify in older adults—e.g. Figs. S3 U,V,W,X
Metatheria Marsupialia Dasyuridae	*Dasyurus spp.* and relatives (Reese et al., 2001)	Extant, Australia	1	Dasyurids (marsupial rat, cat, Tasmanian devil, quoll, etc)
Metatheria Marsupialia Vombatiformes	*Diprotodon spp.*	Pleistocene, Australia	0	Giant, elephant-sized wombatids (extinct)- patella possibly unossified; see Fig. S2 K,L
Metatheria Marsupialia Vombatiformes Vombatidae	*Lasiorhinus latifrons* (Reese et al., 2001)	Extant, Australia	1	Southern hairy-nosed wombat
Metatheria<br>Marsupialia<br>Burramyidae	*Acrobates pygmaeus, Burramys parvus* (Reese et al., 2001)	Extant, Australia	1	Feathertail glider and mountain pygmy possum
Metatheria<br>Marsupialia<br>Phalangeridae	*Trichosurus Vulpecula* (Reese et al., 2001)	Extant, Australia	1	Brush-tailed possum
Metatheria<br>Marsupialia<br>Pseudocheiridae	*Pseudocheirus peregrinus* (Reese at al., 2001)	Extant, Australia	1	Common ringtailed possum
Petauridae	*Petaurus* (Slade, 1810)	Extant, Australia, Indonesia and islands nearby	0	Absence of bony patella

Caluromyidae	*Caluromys* and relatives (Flores, 2009)	Extant, Central and South America	0	Absence of bony patella
Metatheria Marsupialia Vombatiformes Phascolarctidae	*Phascolarctus cinereus* (Reese et al., 2001)	Extant, Australia	1	Koala
Metatheria Marsupialia Peramelidae	*Isoodon obesulus* (Reese et al., 2001)	Extant, Australia	2	Southern bandicoot<br><br>Has patelloid ("suprapatella") also
Metatheria Marsupialia Peramelidae	*Perameles nasuta* (Reese et al., 2001)	Extant, Australia	2	Long-nosed bandicoot<br><br>Has patelloid ("suprapatella") also
Metatheria Marsupialia Notoryctidae	*Notoryctes typhlops* (Johnson & Walton, 1989; Osgood, 1921; Warburton, 2006)	Extant, Australia	2	Marsupial mole
Metatheria Marsupialia Notoryctidae	*Notoryctes caurinus* (Osgood, 1921; Warburton, 2006; Warburton et al., 2003)	Extant, Australia	2	Marsupial mole
Metatheria Marsupialia Microbiotheriidae	*Dromiciops gliroides* (Szalay et al., 2001)	Extant, South America	0	Monito del monte, little bush monkey
Metatheria Marsupialia Caenolestida	*Palaeothentes* spp. (Abello & Candela, 2010)	Miocene, South America	?	Incomplete hindlimb material
Metatheria Marsupialia Caenolestida	*Rhyncholestes raphanurus* (Szalay et al., 2001)	Extant, South America	2	Long-nosed shrew opossum
Metatheria Marsupialia Caenolestida	*Caenolestes fulginosus* (Osgood, 1921)	Extant, South America	2	Dusky shrew opossum
Metatheria Marsupialia Tarsipidae	*Tarsipes rostratus* (Osgood, 1921; Waterhouse, 1846)	Extant, Australia	2	Honey possum
Eutheria	*Asioryctes nemegetensis* (Wible et al., 2005)	Late Cretaceous, Mongolia	?	Basal eutherian but not placental per O'Leary et al. (2013)
Eutheria	*Ukhaatherium nessovi* (Horovitz, 2003)	70-75 Mya Late Cretaceous, Mongolia	2	Basal eutherian but not placental per O'Leary et al. (2013)

Eutheria	*Zalambdalestes lechei* (Wible et al., 2005)	70-75 Mya Late Cretaceous, Mongolia	2	Basal eutherian but not placental per O'Leary et al. (2013)
Eutheria Taeniodonta	*Onychodectes tisonensis* (Matthew, 1937; Schoch, 1986)	Paleocene, USA	2	
Eutheria Taeniodonta	*Psittacotherium multifragum* (Schoch, 1986)	Paleocene, USA	2	
Eutheria Taeniodonta	*Stylinodon mirus* (Schoch, 1986)	Paleocene (but elsewhere described as Eocene)	2	
Eutheria Pantodonta	*Bemalambda* spp. (Chow et al., 1977)	Paleocene, China	2	
Eutheria Pantodonta	Pantolambdidae spp. (Chow et al., 1977; Simons, 1960)	Paleocene, USA	2	
Eutheria Pantodonta	*Coryphodon* sp. (Cope, 1883)	Paleocene, USA	2	
Eutheria Pantodonta	*Titanoides* sp. (Patterson, 1934; Simons, 1960)	Paleocene, USA	2	
Eutheria Apatemyidae	*Apatemys (Teilhardella) chardini* (Koenigswald et al., 2005)	Eocene, USA	2	
Eutheria Notoungulata	*Allalmeia atalaensis* (Lorente et al., 2014)	Eocene, Argentina	2	South American notoungulate
Eutheria Notoungulata	*Toxodon* sp. (Schockey, 2001)	Pliocene/Pleistocene, South America	2	Notoungulate; see Fig. S2 G,H
Eutheria Dinocerata	*Prodinoceras* sp. (Gunnell, 2008)	Paleocene/Eocene, USA	2	
Eutheria Dinocerata	*Uintatherium anceps* (Wheeler, 1961)	Eocene, USA	2	See Fig. S1 W,X
Eutheria Proterotheriidae	*Eoauchenia* sp. (Shockey, 2001)	Miocene/Pliocene, Argentina	2	Early litoptern
Eutheria Xenarthra Cingulata	*Trachycalyptus* sp. (Shockey, 2001)	Miocene, Argentina	2	Or related glyptodonts; e.g. *Panochthus*, Fig. S2 I. Extinct ground sloths, e.g. *Scelidodon,* likewise have a patella—Fig. S2 J; as do extant

				Xenarthra; Fig. S3
Eutheria Afrotheria Embrithopoda	*Arsinoitherium zitteli* (Andrews, 1906)	Eocene/Oligocene, northern Africa to Asia	2	Large relative to elephants, sirenians. See Fig. S1 UV
Eutheria Leptictidae	*Leptictidium nasutum* (Von Koenigswald et al., 1992)	Eocene, Germany	2	Visible in photo
Eutheria Afrotheria Paenungulata	*Carodnia vieirai* (Couto, 1952; O'Leary et al., 2013)	Paleocene/Eocene, Brazil	2	Ungulate, patella marked as present in O’Leary et al. (2013) Morphobank study 773
Eutheria Afrotheria Desmostylia	*Paleoparadoxia* sp. (Yanofsky, 1998)	Miocene, widespread: Japan, North America	2	Renamed (Domning & Barnes, 2007); aquatic mammal
Eutheria Afrotheria Desmostylia	*Desmostylus hesperus* (Inuzuka, 2009)	Oligocene-Miocene, widespread: Japan, Russia, North America	2	Aquatic mammal
Eutheria Afrotheria Sirenia	*Protosiren* sp. (Zalmout, 2008)	Eocene, widespread	2	Early sirenian with hindlimbs from Egypt
Eutheria Afrotheria Sirenia	*Pezosiren portelli* (Domning, 2001)	Eocene, Jamaica	2	Early sirenian with hindlimbs from Jamaica
Eutheria Afrotheria Proboscidea	*Numidotherium koholense* (Court, 1994)	Eocene, northern Africa	?	Hindlimb material lacking
Eutheria Afrotheria Proboscidea	*Moeritherium* sp. (Delmer et al., 2006; O'Leary et al., 2013)	Eocene, northern Africa	2	Marked as present, with photograph, in O’Leary et al. (2013) Morphobank study 773
Eutheria Glires	*Rhombomylus turpanensis* (Meng et al., 2003; O'Leary et al., 2013)	Eocene, China, Mongolia	2	Side branch related to early rodents
Eutheria Glires Rodentia Paramyidae	*Pseudotomus eugenei* (Dunn & Rasmussen, 2007; Dunn, 2009)	Eocene, North America	2	
Eutheria Glires Rodentia Paramyidae	*Paramys delicates* (Matthew, 1910; Wood, 1962)	Paleocene-Oligocene, widespread	2	
Eutheria Glires Rodentia Sciurognathi	*Ischyromys* sp. (Scott et al., 1937)	Eocene-Oligocene, North America	2	
Eutheria Glires Rodentia	*Nonanomalurus soniae* (Pickford	Oligocene, Morocco	2	

	et al., 2013)			
Eutheria Primates Haplorhini	*Teilhardina belgica* (Gebo et al., 2012)	Eocene, Belgium, France	?	Hindlimb material incomplete
Eutheria Plesiadapiformes	*Plesiadapis cookei* (Bloch & Boyer, 2007)	Paleocene, USA	2	
Eutheria Primates Notharctidae	*Notharctus* spp. (Gregory, 1920)	Eocene, USA	2	Fig. S1 S,T
Eutheria Primates Notharctidae	*Darwinius masillae* (Franzen et al., 2009)	Eocene, Germany	2	
Eutheria Primates Notharctidae	*Pelycodus jarrovii* (Cope, 1885)	Eocene, USA	2	
Eutheria Primates Haplorhini	*Archicebus achilles* (Ni et al., 2013)	Eocene, China	2	
Eutheria Primates Haplorhini Omomyidae	*Omomys* sp. (Anemone & Covert, 2000)	Eocene, North America	2	
Eutheria Primates Haplorhini	*Pliopithecus* sp. (Zapfe, 1958)	Miocene, Eurasia	2	
Eutheria Primates Hominoidea	*Oreopithecus* sp. (Schoch, 1986)	Miocene, Italy, Moldova	2	
Eutheria Primates Hominoidea	*Proconsul* spp. (Ward et al., 1995)	Oligocene-Miocene, Kenya	2	
Eutheria Primates Hominoidea	*Nacholapithecus kerioi* (Nakatsukasa et al., 2012)	Miocene, Kenya	2	
Eutheria Primates Hominoidea	*Pierolapithecus catalaunicus* (Pina et al., 2014)	Miocene, Spain	2	
Eutheria Primates Hominidae	*Australopithecus afarensis* (Alemseged et al., 2006)	Pliocene, Ethiopia, Kenya	2	"Lucy" hindlimb incomplete, patella noted in juvenile, est. 3 years old
Eutheria Primates Hominidae	*Australopithecus robustus* (Susman, 1988)	Pliocene/Pleistocene, South Africa	2	Previously *Paranthropus robustus*
Eutheria Primates Hominidae	*Homo* spp. (Trinkhaus, 2000)	850, 300, 150 Kya, Pleistocene/Extant, Africa/Eurasia	2	
Eutheria Pholidota	*Eomanis waldi*	Eocene, Germany	2	Early pangolin

	(Storch & Richter, 1992)			
Eutheria Palaeanodonta	*Ernanodon antelios* (Kondrashov & Agadjanian, 2012)	Paleocene, China, Mongolia	2	
Eutheria Palaeanodonta	*Brachianodon westorum* (Gunnell & Gingerich, 1993)	Eocene, USA	2	
Eutheria Creodonta Hyaenodontidae	*Arfia shoshoniensis* (Gingerich & Deutch, 1989)	Eocene, USA	2	Side branch to extant carnivores
Eutheria Creodonta Oxyaenidae	*Oxyaena* sp., *Patriofelis* sp. (Osborn, 1900; Wortman, 1894)	Paleocene-Eocene, widespread	2	Side branch to extant carnivores
Eutheria Carnivoramorpha	*Paroodectes feisti* (Springhorn, 1992)	Eocene, Germany	2	Visible in photo; side branch to extant carnivores
Eutheria Carnivoramorpha	*Miacis* spp. (Spaulding & Flynn, 2012)	Eocene, widespread	?	Incomplete hindlimb material
Eutheria Carnivoramorpha	*Vulpavus ovatus* (O'Leary et al., 2013; Spaulding et al., 2012)	Eocene, USA	2	Marked as present, with photo, in O'Leary et al. (2013) Morphobank study 773
Eutheria Carnivora Nimravidae	*Hoplophoneus primaevus* (Burnham et al., 2013)	Oligocene, USA	2	Fig. S1 K,L
Eutheria Carnivora Nimravidae	*Pogonodon brachyops* (Cope, 1883)	Oligocene, USA	2	
Eutheria Carnivora Mustelidae	*Brachypsalis modicus* (Galbreath, 1955)	Miocene, USA	2	
Eutheria Chiroptera	*Icaronycteris index* (Jepsen, 1966; O'Leary et al., 2013)	Eocene, USA	2	Early bat, patella marked as present, with photograph, in O'Leary et al. (2013) Morphobank study 773
Eutheria Chiroptera	*Tachypteron franzeni* (Storch et al., 2002)	Eocene, Germany	2	Early bat
Eutheria Chiroptera (O'Leary et al.,	*Onychonycteris finneyi* (O'Leary	Eocene, USA	2	Early bat, patella marked as present, with photograph, in O'Leary et al.

2013)	et al., 2013)			(2013) Morphobank study 773
Eutheria Microchiroptera	*Microchiroptera* spp. (Smith et al., 1995)	Extant	2	Bats
Eutheria Microchiroptera	*Lasiurus cinereus* (Czaplewski et al., 2008)	Extant	2	Hoary bat
Eutheria Megachiroptera	*Cynopterus marginatus* (Pearson & Davin, 1921)	Extant	2	Lesser short-nosed fruit bat
Eutheria Megachiroptera	*Rousettus aegypticus* (Smith et al., 1995)	Extant	2	Egyptian fruit bat
Eutheria Megachiroptera	*Cynopterus brachyotis* (Smith et al., 1995)	Extant	2	Lesser short-nosed fruit bat
Eutheria Megachiroptera	*Haplonycterus fischeri* (Smith et al., 1995)	Extant	2	Fischer's pygmy fruit bat
Eutheria Megachiroptera	*Pteropus* spp. (Smith et al., 1995)	Extant	0	"Flying foxes"
Eutheria Condylarthra(?)	*Protungulatum donnae* (Archibald et al., 2011)	Paleocene, North America	?	Incomplete postcrania
Eutheria Condylarthra	*Arctocyon primaevus* (Argot, 2013)	Paleocene, France	2	
Eutheria Condylarthra	*Mioclaenus* sp. (Cope, 1883; Matthew, 1937; Schlosser, 1887)	Paleocene, USA	2	
Eutheria Condylarthra	*Phenacodus* spp. (Cope, 1883; Osborn, 1898; Rose et al., 2012; Thewissen, 1990)	Paleocene-Eocene, widespread	2	
Eutheria Condylarthra	*Copecion brachypternus* (Thewissen, 1990)	Paleocene-Eocene, USA	2	
Eutheria Condylarthra	*Tetraclaenodon puercensis* (Kondrashov & Lucas, 2012;	Paleocene-Eocene, North America	2	Originally named "*Euprotogonia*"; patella reported in Eocene but not Paleocene specimen, earlier hindlimb material incomplete

	Matthew, 1897)			
Eutheria Condylarthra	*Chriacus* sp. (Rose, 1987)	Paleocene-Eocene, North America	2	
Eutheria Condylarthra	*Meniscotherium* sp. (Cope, 1883; Guzin, 1965)	Eocene, North America	2	
Eutheria Condylarthra	*Hyopsodus paulus* (O'Leary et al., 2013)	Eocene, USA	2	Marked as present, including photo, in O'Leary et al. (2013) Morphobank study 773
Eutheria Mesonychia	*Pachyaena gracilis* (O'Leary & Rose, 1995)	Eocene, USA	2	
Eutheria Mesonychia	*Pachyaena ossifraga* (Zhou et al., 1992)	Eocene, USA, United Kingdom	2	
Eutheria Mesonychia	*Mesonyx obtusidens* (Cope, 1873; Cope, 1883; O'Leary et al., 2013)	Eocene, USA	2	
Eutheria Perissodactyla Chalicotheriidae	*Litolophus gobiensis* (Bin, 2009; Warburton, 2004)	Eocene, China	2	
Eutheria Perissodactyla Palaeotheriidae	*Hyracotherium granger* (Cope, 1883; Danaher et al., 2009; Williamson et al., 2012; Wood et al., 2011)	Eocene, widespread	2	Stem equid; or "*Eohippus*" (controversial taxonomy)
Eutheria Perissodactyla	*Hyrachyus* sp. (Danaher et al., 2009)	Eocene-Oligocene, widespread	2	
Eutheria Perissodactyla Equidae	*Miohippus* spp. (formerly *Mesohippus* etc.) (Sinclair, 1925; O'Leary et al., 2013; Zheng et al., 2013)	Eocene-Miocene, North America	2	Fig. S1 A,B
Eutheria Perissodactyla Equidae	*Propalaeotherium hassiacum* (Franzen, 1992)	Eocene, Germany	2	Originally considered a palaeotherid, re-interpreted as early equid (Abello *et al.*, 2010)
Eutheria Perissodactyla Equidae	*Archaeohippus ultimus* (Sullivan, 2003)	Miocene, North America	2	

Eutheria Perissodactyla Equidae	*Equus simplicdens* (was *Plesihippus shoshonensis*) (Gazin, 1936)	Pliocene-Pleistocene, North America	2	Also called Hagerman's horse
Eutheria Perissodactyla Rhinocerotidae	*Subhyracodon mitis* (Pina et al., 2014)	Eocene-Oligocene, USA	2	Stem rhinoceros
Eutheria Perissodactyla Rhinocerotidae	*Paraceratherium* spp. (Danaher et al., 2009; Mitchell et al., 2014; Osborn, 1921)	Eocene-Oligocene, Turkey, Pakistan, Kazakhstan, Mongolia, China	2	Also called "*Indricotherium*" or "*Baluchitherium*", taxonomy not fully resolved. Giant stem rhinoceros
Eutheria Perissodactyla Brontotheriidae	*Palaeosyops paludosus* (Leidy, 1873)	Eocene, USA	2	Rhinoceros-like
Eutheria Perissodactyla Brontotheriidae	*Aktautitan hippopotamopus* (Mihlbachler et al., 2004)	Eocene, Kazakhstan	2	Rhinoceros-like
Eutheria Perissodactyla Brontotheriidae	*Embolotherium andrewsi* (McLaughlin et al., 2010; Meredith et al., 2009)	Eocene, China, Mongolia	2	Rhinoceros-like
Eutheria Artiodactyla	*Diacodexis metsiacus* (Alemseged et al., 2006; Rose, 1985)	Eocene, USA	2	Stem artiodactyl
Eutheria Artiodactyla	*Protoreodon annectens* (Ludtke, 2007)	Eocene, North America	2	
Eutheria Artiodactyla	*Indohyus* sp. (Cooper et al., 2012)	Eocene, China, India, Pakistan	2	Closely related to early cetaceans
Eutheria Artiodactyla	*Gervachoerus jaegeri* (Erfurt & Metais, 2007)	Eocene, France, Germany	2	
Eutheria Artiodactyla Anthracotheriidae	*Elomeryx armatus* (Yuan et al., 2013)	Oligocene, North America	2	
Eutheria Artiodactyla	*Leptomeryx evansi* (Schultz, 1960)	Eocene-Oligocene, North America	2	Patella documented in widely diverse (Cet)Artiodactyla—e.g. Fig. S3 S,T in juvenile *Hippopotamus*, diminutive *Tragulus*
Eutheria Artiodactyla	*Caenotherium* sp. (Erfurt et al.,	Eocene-Miocene, Europe	2	

	2007)			
Eutheria Artiodactyla Ruminantia Camelidae	*Genticamelus* (*Paratylopus*) *sternbergi* (Cope & Matthew, 1915)	Oligocene, USA	2	
Eutheria Cetartiodactyla Artiodactyla Merychoidodontidae	*Ustatochoerus (Merychyus) major* (Morgan et al., 2009)	Miocene, USA	2	Cf. Fig. S1 C,D
Eutheria Cetacea	*Pakicetus attocki* (Thewissen et al., 2001)	Eocene, India, Pakistan	?	Very early cetacean with hindlimbs, specimens incomplete but often shown with patella (Fig. S1 M,N)
Eutheria Cetacea	*Maiacetus inuus* (Gingerich et al., 2009)	Eocene, Pakistan	2	Early cetacean with hindlimbs
Eutheria Cetacea	*Basilosaurus isis* (Gingerich et al., 1990)	Eocene, northern Africa	2	Early cetacean with hindlimbs
Eutheria Cetacea	*Ambulocetus natans* (Madar et al., 2002)	Eocene, Pakistan	2	Early cetacean with hindlimbs. Fig. S1 O,P
Eutheria Cetacea	*Rodhocetus balochistanensis* (Gingerich et al., 2009)	Eocene, Pakistan	2	Early cetacean with hindlimbs
Eutheria Cetacea	*Artiocetus clavis* (O'Leary et al., 2013)	Eocene, Pakistan	2	Early cetacean with hindlimbs, Marked as present in O'Leary et al. (2013) Morphobank study 773
Eutheria Cetacea	*Dorudon atrox* (Uhen, 2004)	Eocene, northern Africa	2	Early cetacean with very reduced hindlimbs. Fig. S1 Q,R

[$] Early groups are ordered by estimated times of divergence, not by geological age of representative specimens. The Eutheria specifically are ordered as per Figure 7 (from bottom to top), thus by major clades (mostly "orders") and then by geological age of specimens within the major taxa.

[#] Common names in some cases from general sources.

[%] "Condylarthra" probably does not represent a monophyletic group but this term is widely used in the literature and is generally understood (Rose, 2006).

Supplementary Table S2. Genetics of the patella in humans

Syndrome	Gene	OMIM ID#	Molecular function	Developmental Process
Nail-patella	LMX1B	602575	Transcription factor	Limb patterning
Small patella	TBX4	601719	Transcription factor	Limb patterning
Clubfoot with long bone deficiencies	PITX1	602149	Transcription factor	Limb patterning
Fuhrmann	WNT7A	601570	Cell-cell signalling	Limb patterning
Du Pan	GDF5	601146	Growth factor	Bone growth and differentiation, joint definition/patterning
Multiple pterygium (Escobar variant)	CHRNG	100730	Fetal muscle cholinergic nicotinic receptor	Neuromuscular synaptic signalling
Multiple epiphyseal dysplasia	SL26A2, COL9A2	606718,120260	Sulfate transporter, collagen	Chemistry of connective tissue
Meier-Gorlin	ORC1, ORC4, ORC6, CDT1, CDC6, GMNN	601902,603056, 607213,605525, 602627,602842	Origin recognition binding	DNA replication, cell division
Roberts	ESCO2	609353	Sister chromatid cohesion acetyltransferase	DNA replication, cell division
RAPADILINO	RECQL4	603780	DNA helicase	DNA replication, cell division
Genitopatellar	KAT6B	605880	Histone acetyltransferase	Chromatin
Proteus	AKT1	164730	Phosphoinositide 3-kinase	Cell growth

Supplementary Figures (S1-S6):

Supplementary Figure S1. Patella in representative mammals of the Paleogene (Eocene, Oligocene) and early Neogene (Miocene). Red arrows denote the patella. A, B. *Mesohippus* sp. (Oligocene, early horse). C, D. *Merycoidodon (AKA Oreodon) culbertsoni* (late Eocene/early Miocene pig-like), patella attested for Oligocene *Merycoidodon/Oreodon gracilis* and *Oreodon sp.* (Gilmore, 1906; Leidy, 1869). E, F. *Megacerops sp.* (late Eocene, rhinoceros-like), patella attested for *Megacerops tyleri (Lull, 1905)*. G, H. *Archaeotherium sp.* (cast, Eocene/Oligocene, fanged peccary-like entelodont), patella attested for *Archaeotherium* and related *Daedodon sp.* (originally *Dinohyus*, since renamed) (O'Leary *et al.*, 2013; Peterson, 1909). I, J. *Hyaenodon gigas* (Eocene/Miocene creodont), patella attested (Hill, 1886). K, L. *Hoplophoneus sp.* (Eocene/Oligocene, feline-like nimravid), patella attested (Turner & Anton, 1997). M, N. *Pakicetus attocki* (Eocene, early cetacean), patella not attested by primary citation. O, P. *Ambulocetus natans* (Eocene, aquatic and ambulatory cetacean), patella attested (Madar *et al.*, 2002). Q, *Dorudon atrox* (aquatic cetacean, upper specimen, pelvis and hindlimbs not visible behind *Ambulocetus* lower specimen). R, *Dorudon atrox* patella closeup, patella attested (Uhen, 2004). S, T. *Notharctus sp.* (Eocene, early primate), patella attested (Gregory, 1920). U, V. *Arsinoitherium zitteli* (Eocene/Oligocene, rhinoceros-like afrotherian), patella documented (Andrews, 1906); exhibit specimen at The Natural History Museum (NHMUK), London, United Kingdom. W, X. *Uintatherium anceps* (Eocene, large-bodied member of the exotic stem-placental clade Dinocerata); exhibit specimen at the Galeries d'Anatomie Comparée et de Paléontologie, Paris, France. Specimen numbers (CMNFV = Canadian Museum of Nature, fossil vertebrates): A, B. CMNFV 52723. C, D. CMNFV 8181. E, F. CMNFV 341. G, H. CMNFV 52721. I, J. CMNFV 52756. K, L. CMNFV 52785. M, N. CMNFV 51974. O, P. CMNFV 51838. Q, R. CMNFV 51837. S, T. CMNFV 52972. Exhibit specimens current as of 2015. Cast vs. original fossil material not distinguished here (see also main text).

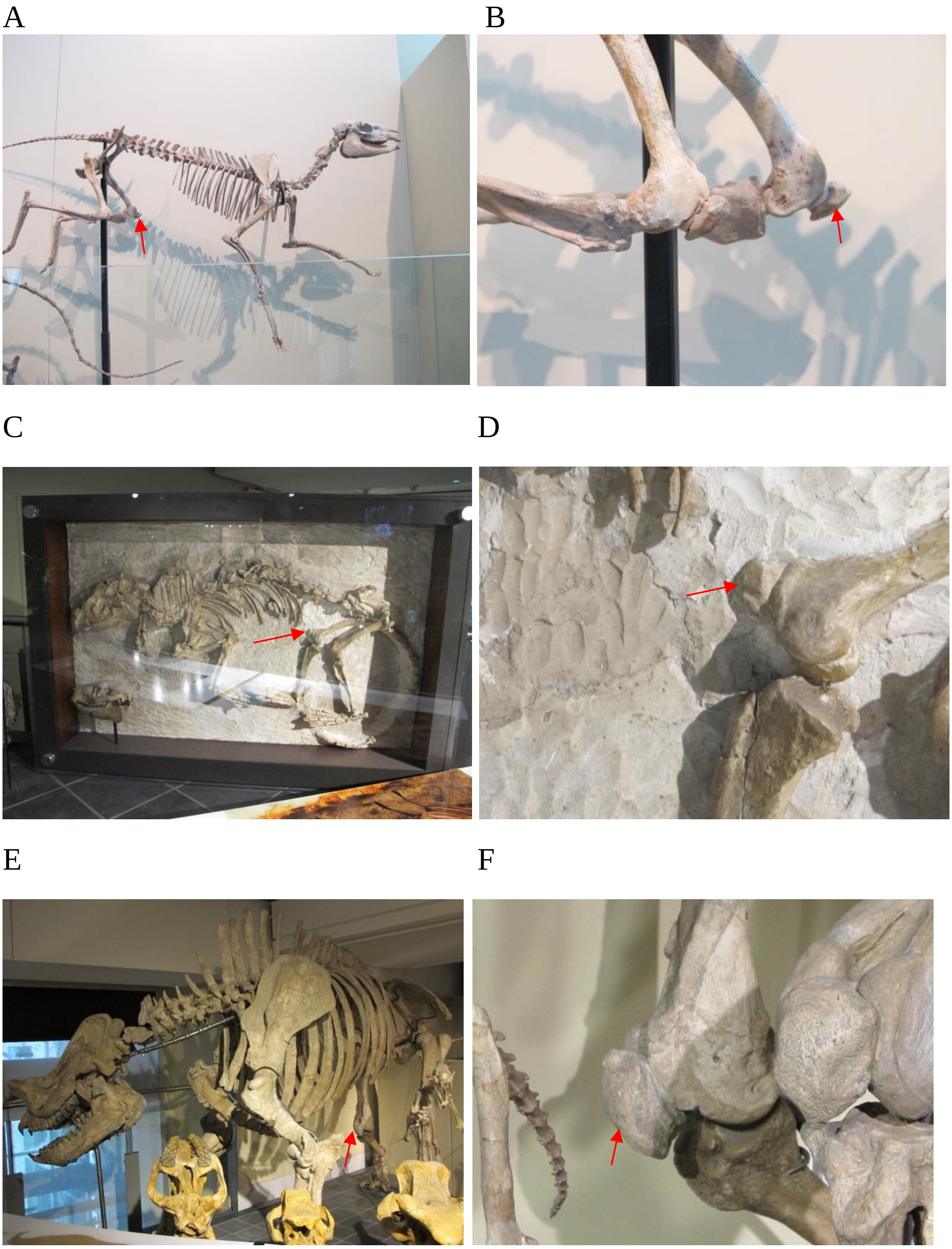
A
B
C
D
E
F

G

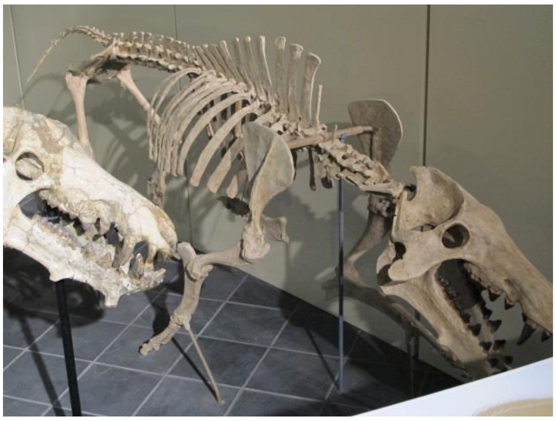

H

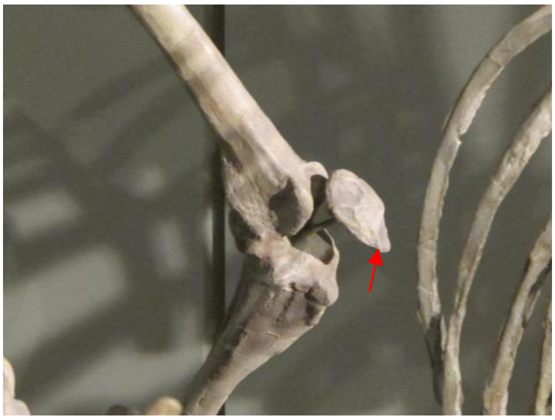

I

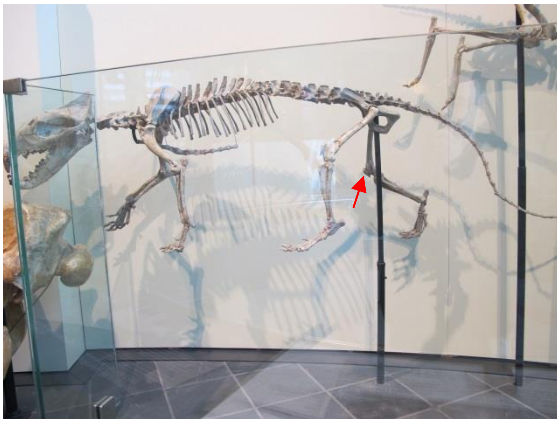

J

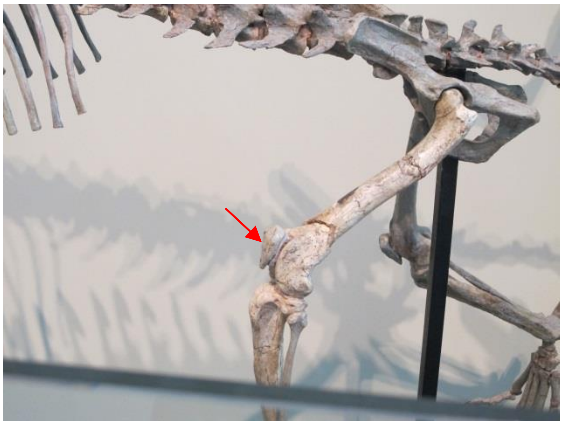

K

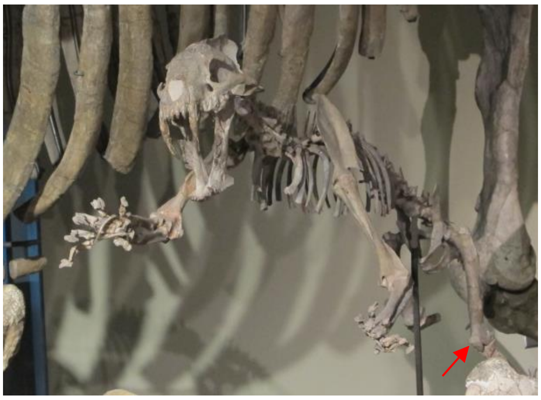

L

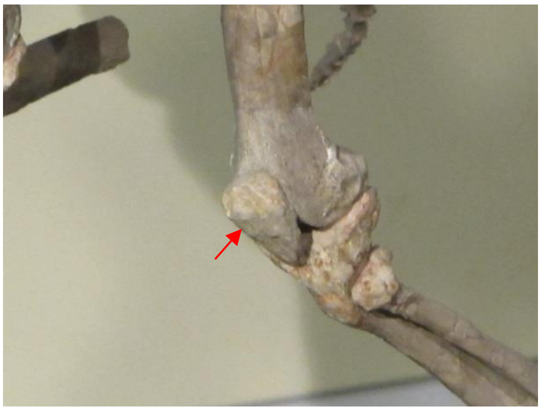

M

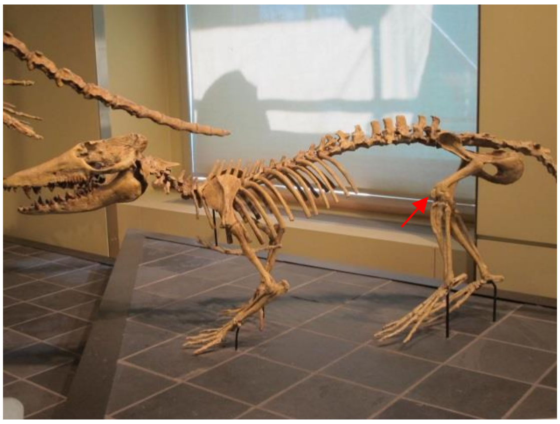

N

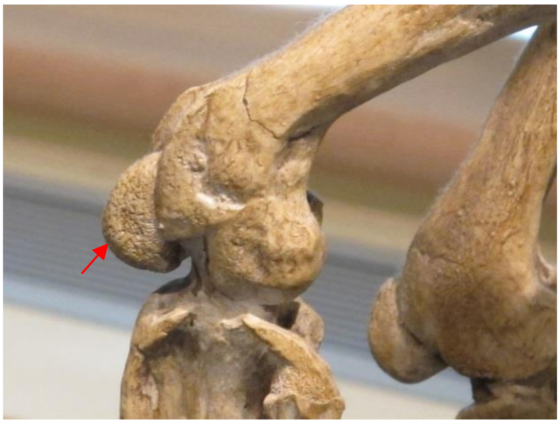

O

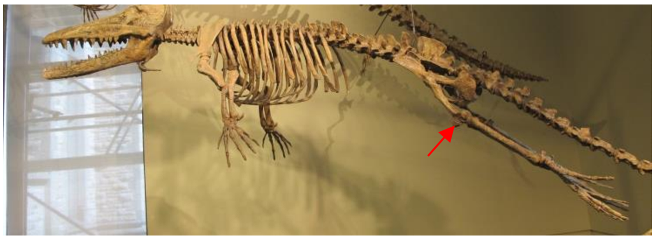

P

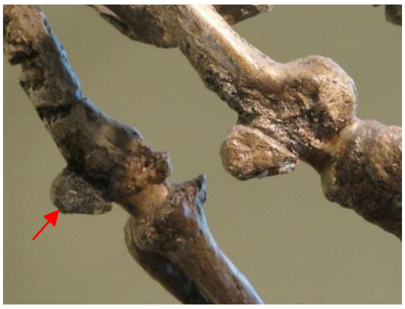

Q

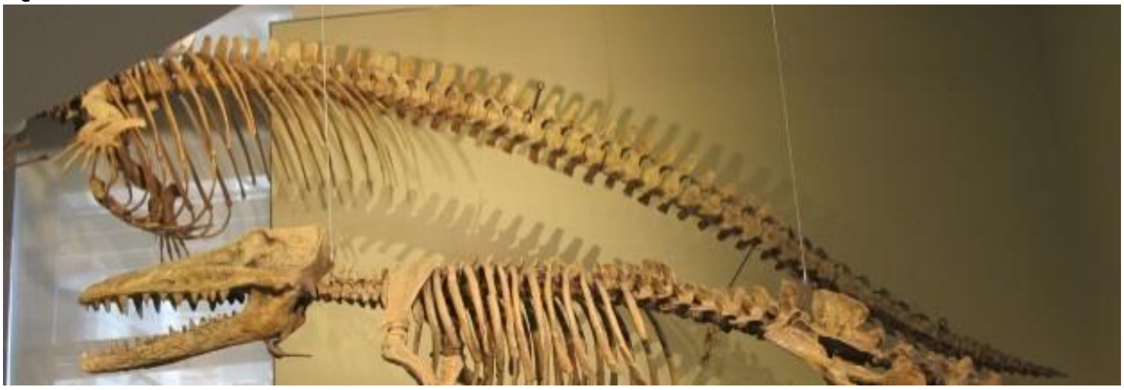

R

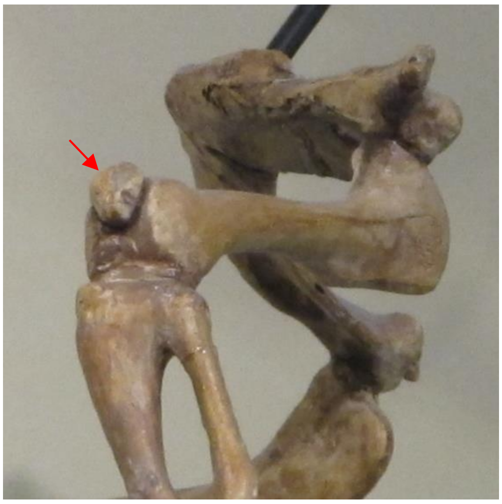

S

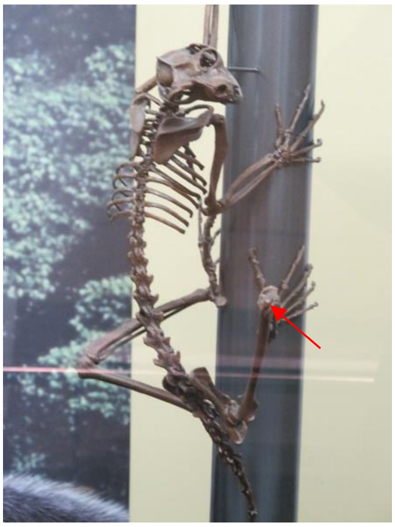

T

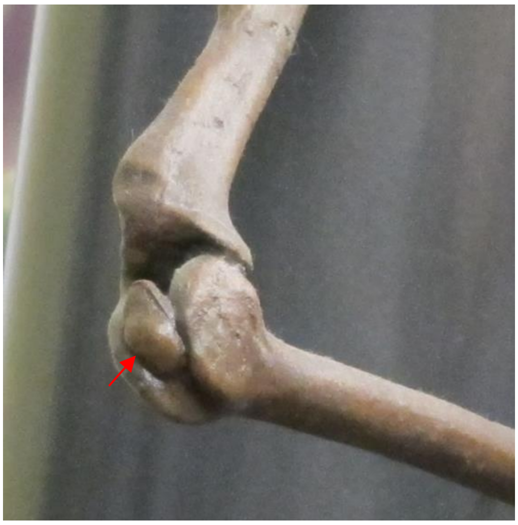

U

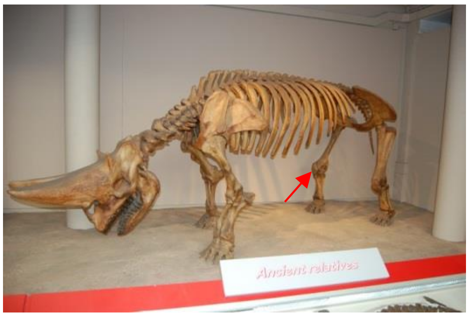

V

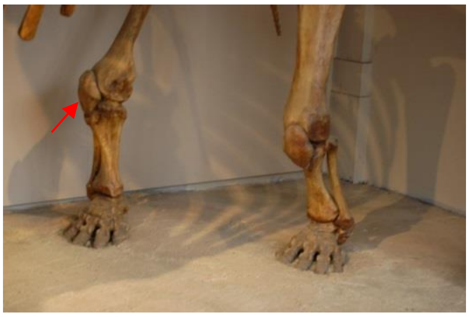

W

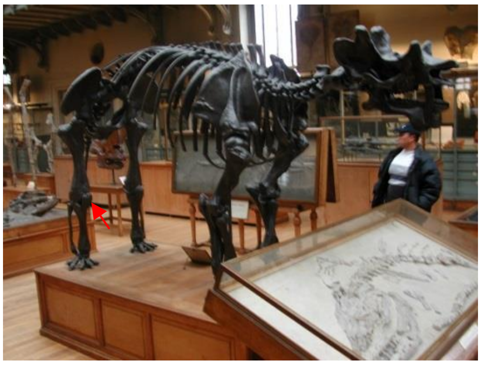

X

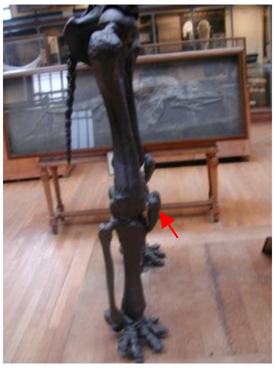

Supplementary Figure S2. Patella in representative mammals of the late Neogene (Pliocene) and early Quaternary (Pleistocene). Red arrows denote the patella. Yellow arrows denote a patellar sulcus (distal groove on the femur) where a patella is absent. A, B. *Castoroides ohioensis* (Pleistocene, giant beaver) (Erickson, 1962). C, D. *Smilodon fatalis* (Pleistocene, sabretooth cat) (Berta, 1987; Raymond & Prothero, 2010). E. *Mammuthus sp.* (Pliocene/mid-Holocene, mammoth, primary collection). F. *Mammut americanum* (Pleistocene, American mastodon) (Woodman & Branstrator, 2008). G, H. *Toxodon* sp. (Plio-Pleistocene, South American notoungulate). I. *Panocthus* sp., glyptodontid (Xenarthra; giant Pleistocene armoured relative of armadillos). J. *Scelidodon* sp. (Xenarthra; giant Pleistocene ground sloth). K, L. *Diprotodon* sp. (giant Pleistocene wombatid marsupial with no evidence of an ossified patella). Specimen numbers: A, B. CMNFV 44757 (CMNFV = Canadian Museum of Nature, fossil vertebrates). C, D. UCMP exhibit specimen (Univ. Museum of Paleontology, Berkeley, California). E. CMNFV 15490; 18994; 19378; 24031; 26771; 27311; 27629; 31645; 32009; 33606; 39632; 43140; 43280; 44321; (1 uncatalogued). F. CMNFV 46768. G, H. exhibit specimen at Museo Paleontológico Egidio Feruglio museum, Trelew, Argentina. I. exhibit specimen at Museo Argentino de Ciencias Naturales “Bernardino Rivadavia” museum, Buenos Aires, Argentina. J. exhibit specimen at Museo Argentino de Ciencias Naturales “Bernardino Rivadavia” museum, Buenos Aires, Argentina. K, L. exhibit specimen at The Natural History Museum (NHMUK), London, United Kingdom. Exhibit specimens current as of 2015. Cast vs. original fossil material not distinguished here (see also main text).

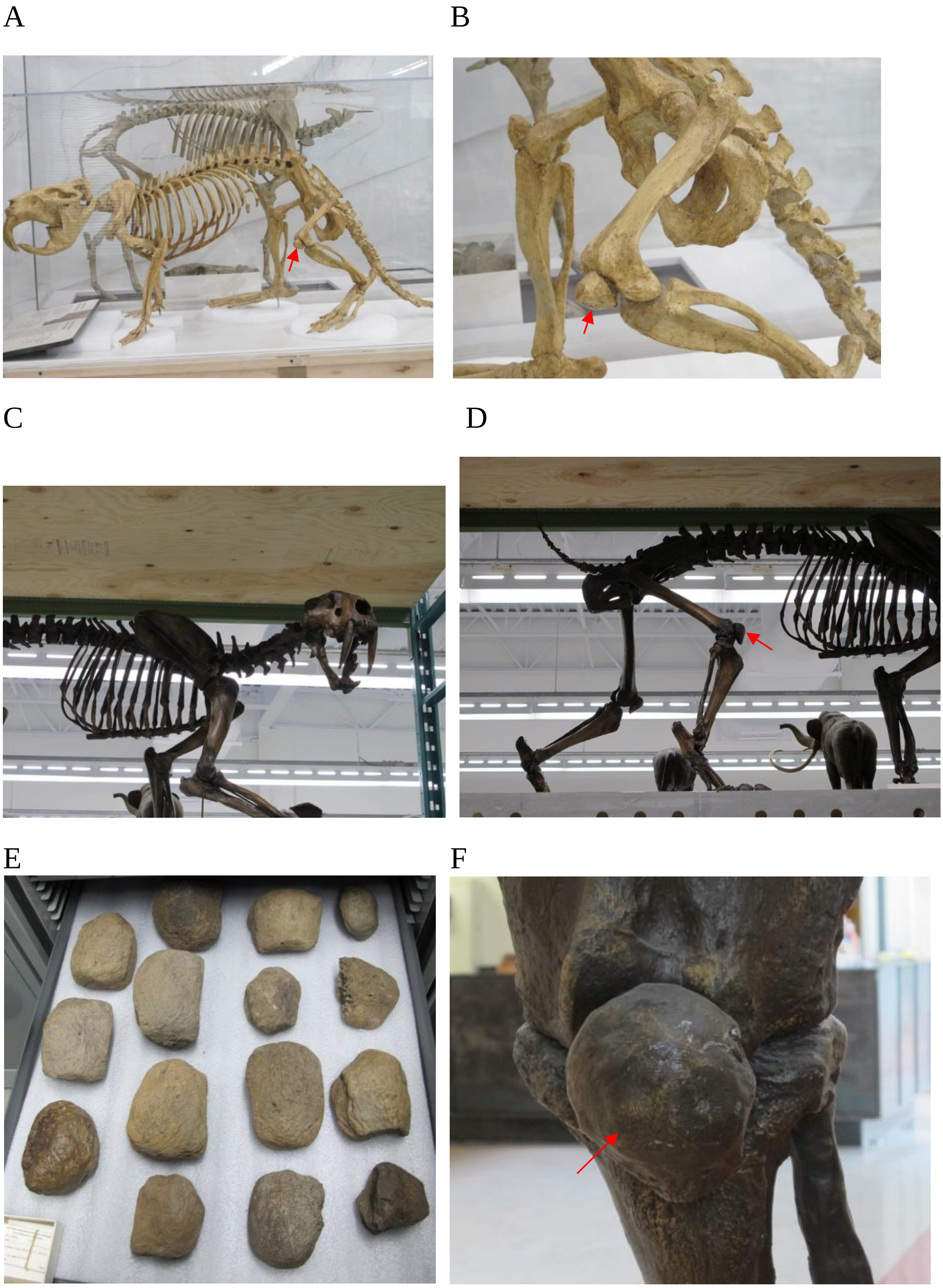

A
B
C
D
E
F

G

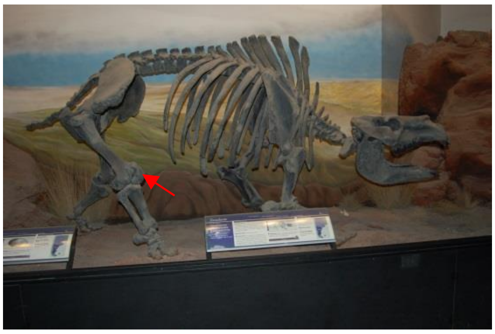

H

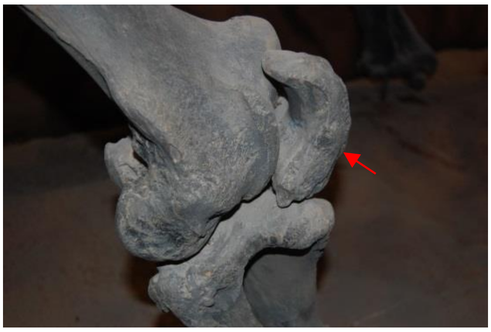

I

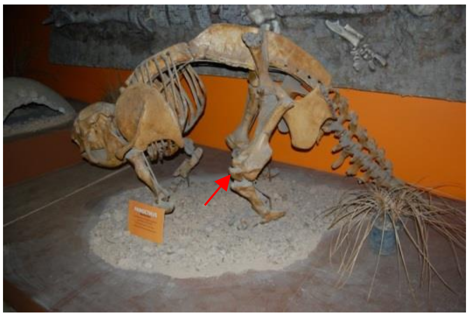

J

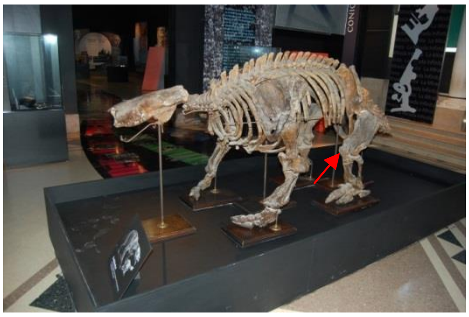

K

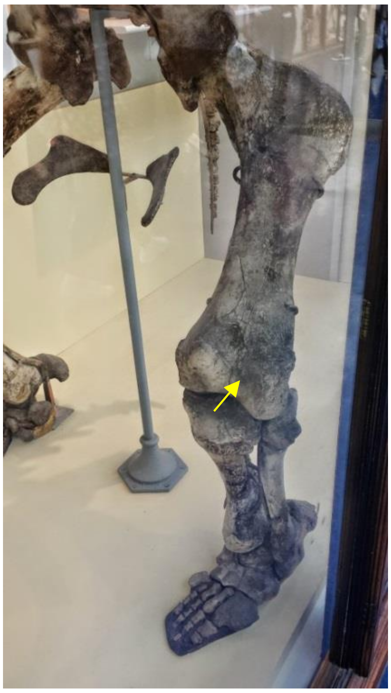

L

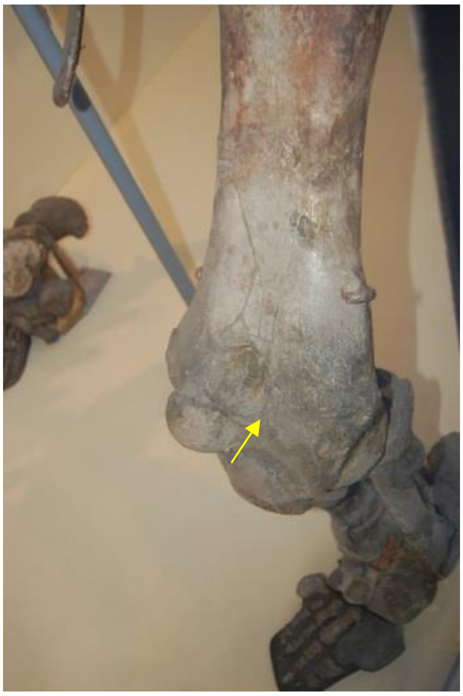

Supplementary Figure S3. Patella in representative extant mammals. Red arrows denote the patella; or unossified tissue of interest (see below and main text). A. *Marmota monax* (woodchuck, rodent). B. *Sciurus carolinensis* (Eastern grey squirrel, rodent). C. *Lepus americanus* (snowshoe hare, lagomorph). D. *Felis catus* (domestic cat, feline carnivore). E. *Canis familiaris* (domestic bulldog, canine carnivore). F. *Phoca vitalina* (harbor seal, pinniped carnivore). G. Suborder *Vermilingua sp.* (anteater, pilose xenarthran). H. *Erinaceus sp.* (hedgehog, eulipotyphlan). I. *Dasypus novemcinctus* (nine-banded armadillo, cingulate xenarthran). J. *Loris lydekkerianus* (grey slender loris, primate). K. *Ursus maritimus* (polar bear, ursine carnivore). L. *Bison bison* (American bison, artiodactyl). M. *Ovibos moschatus* (muskox, artiodactyl). N. *Cervus canadensis* (wapiti, artiodactyl). O. *Alces alces* (moose, artiodactyl). P. *Antilocapra americana* (pronghorn, artiodactyl). Q. *Giraffa camelopardalis* (giraffe, artiodactyl). R. *Rhinoceros unicornis* (Indian rhinoceros, perissodactyl). S. *Hippopotamus amphibius* (artiodactyl), neonatal specimen (with well ossified patella). T. *Tragulus javanicus* (chevrotain, artiodactyl). U, V. *Macropus* sp. (kangaroo, Diprotodontidae, Marsupialia) with possible ossified patellae. W, X. *Onychogalea fraenata* (wallaby, Diprotodontidae, Marsupialia) with possible fibrocartilaginous patellae. Y, Z. *Thylacinus cynocephalus* (thylacine, Thylacinidae, Marsupialia) with possible fibrocartilaginous patellae. Specimen numbers (CMNMA = Canadian Museum of Nature, mammal collection): A. CMNMA 32245. B. CMNMA 11793. C. CMNMA 11795. D. CMNMA (uncatalogued). E. CMNMA 32033. F. CMNMA 32190. G. CMNMA 31911. H. CMNMA 31848. I. CMNMA 31920. J. CMNMA 31894. K. CMNMA (uncatalogued). L. CMNMA 1896. M. CMNMA (uncatalogued). N. CMNMA 1894. O. CMNMA 4850. P. CMNMA (uncatalogued). Q. CMNMA 52859. R. CMNMA 52862. S. on exhibit at Galeries d'Anatomie Comparée et de Paléontologie, Paris, France. T. on exhibit at the University Museum of Zoology, Cambridge, United Kingdom. S,U,V. on exhibit at Museo Argentino de Ciencias Naturales "Bernardino Rivadavia" museum, Buenos Aires, Argentina. W, X. on exhibit at the University Museum of Zoology, Cambridge, United Kingdom. Y, Z. on exhibit at the University Museum of Zoology, Cambridge, United Kingdom. Exhibit specimens current as of 2015. Cast vs. original fossil material not distinguished here (see also main text).

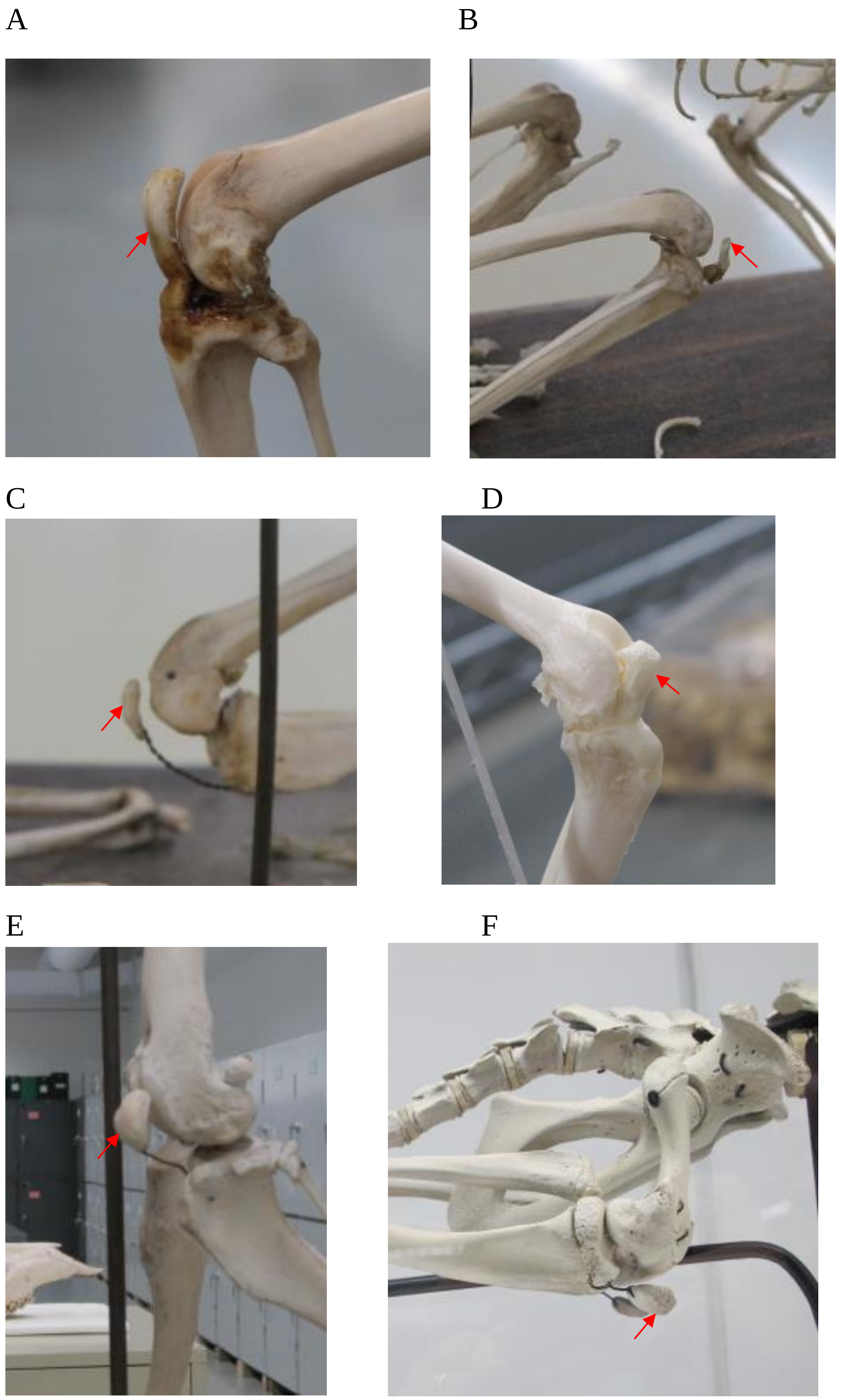
A
B
C
D
E
F

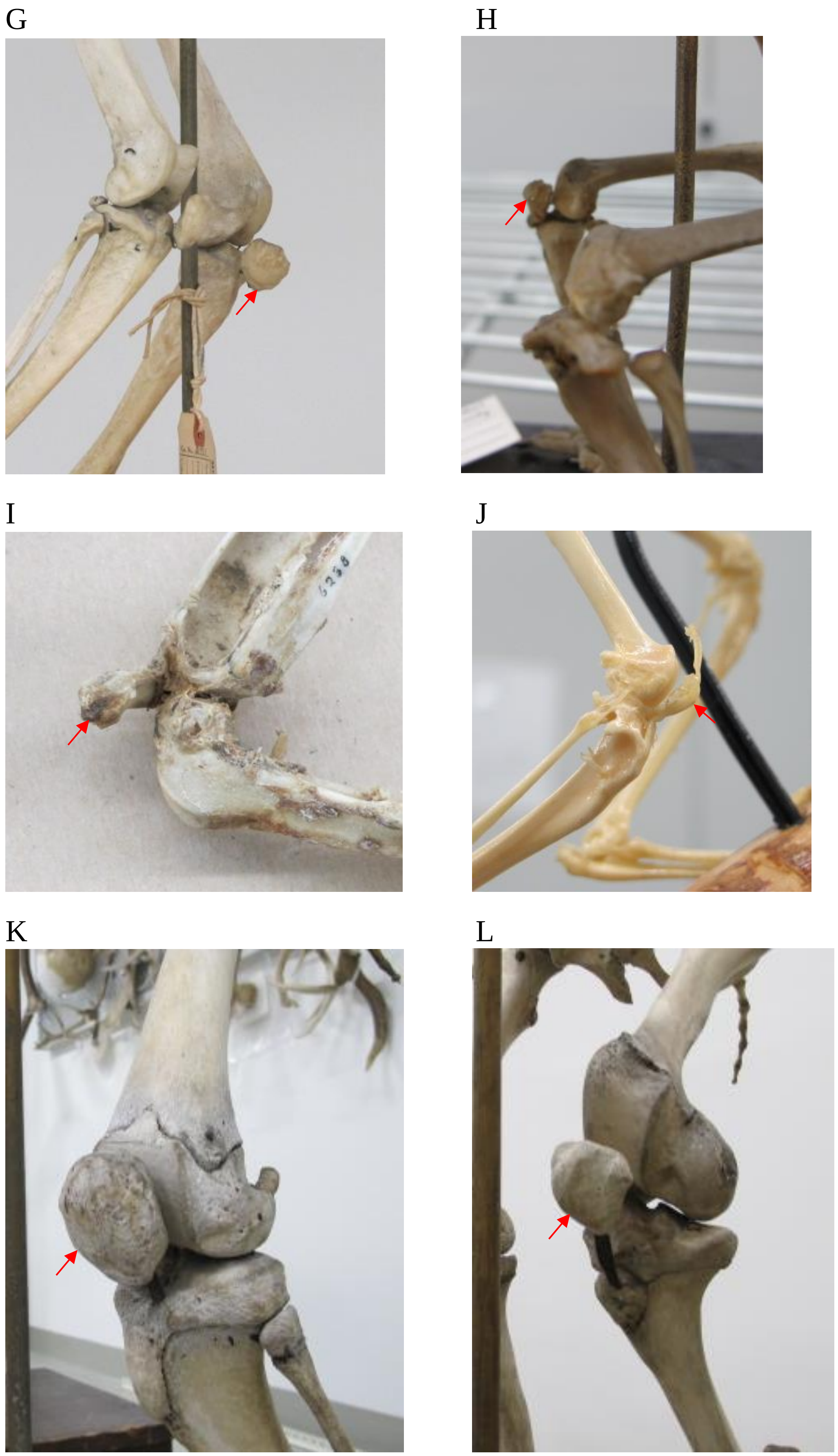
G
H
I
J
K
L

M

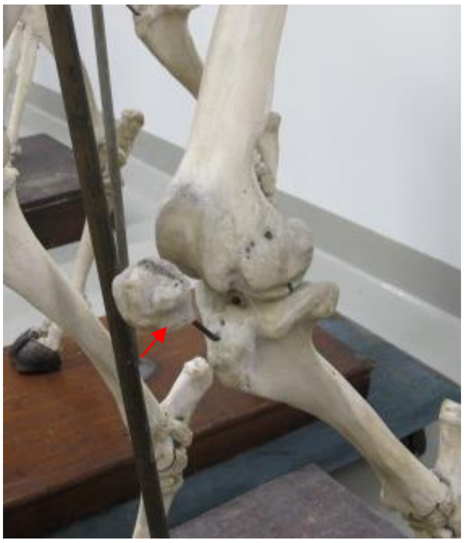

N

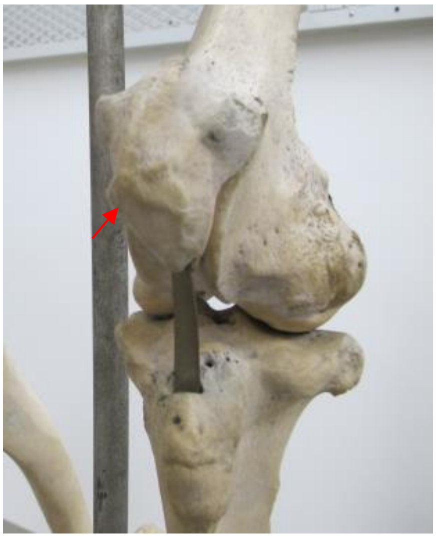

O

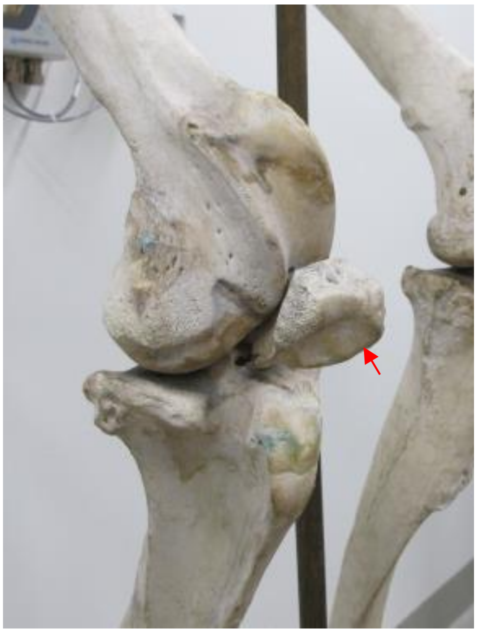

P

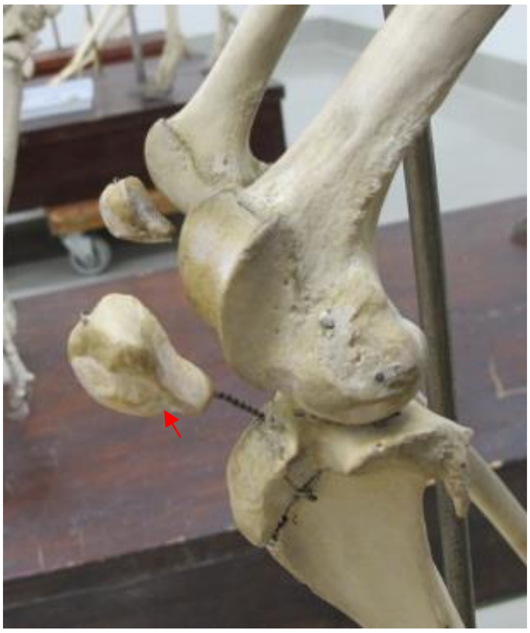

Q

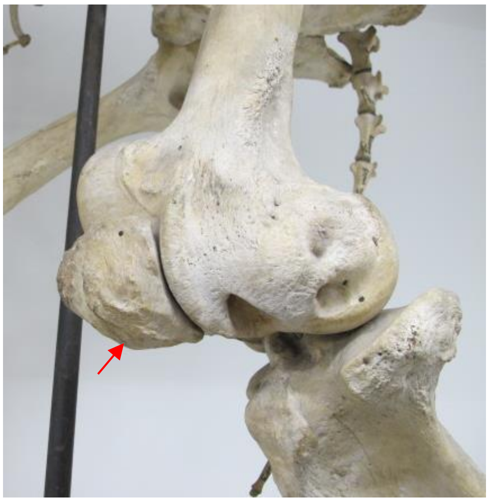

R

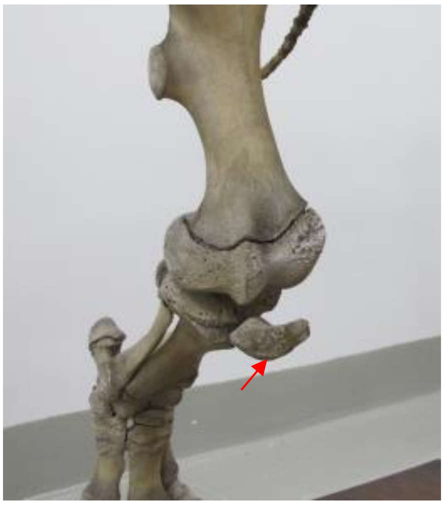

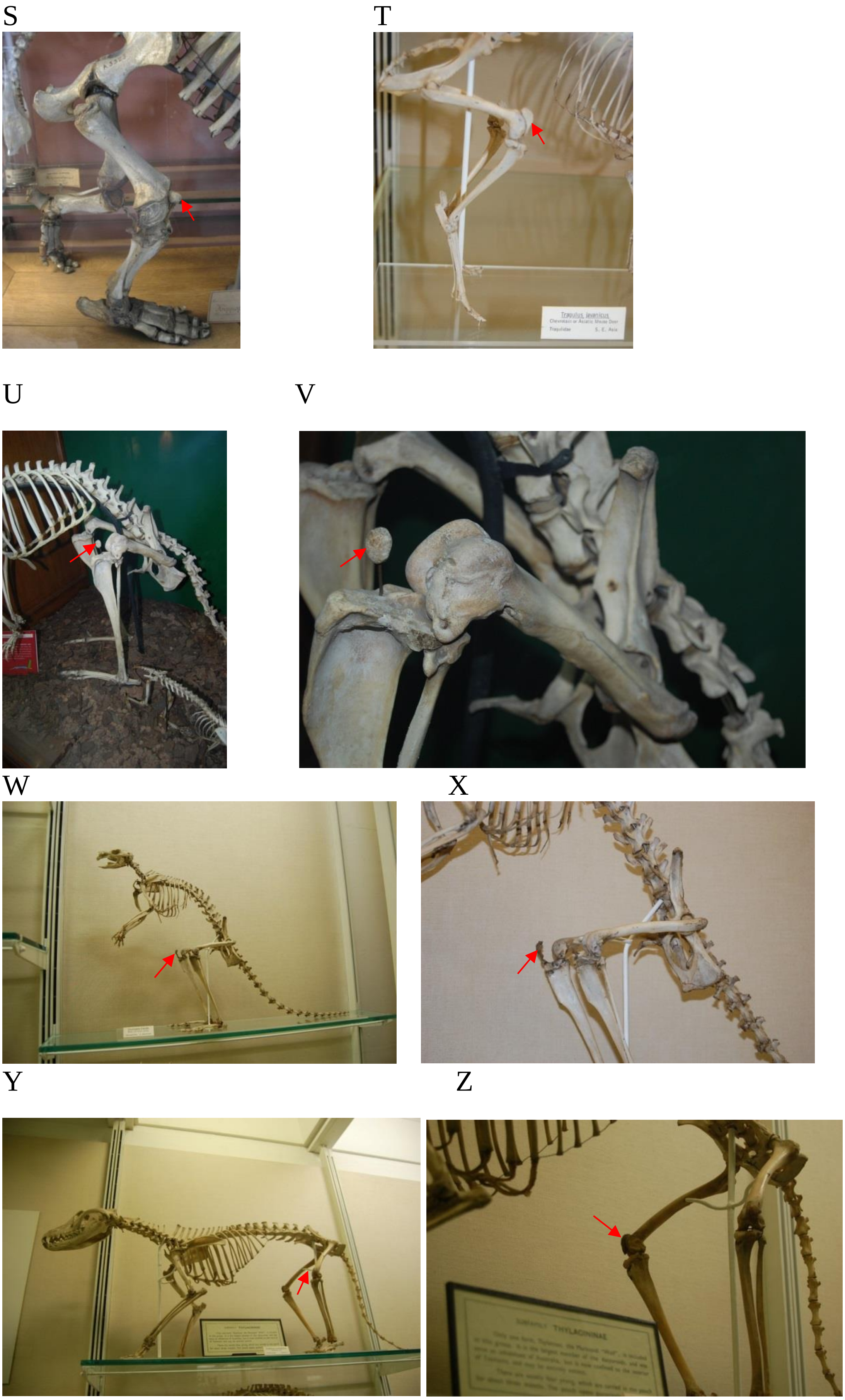
S
T
U
V
W
X
Y
Z

Supplementary Figure S4. Ancestral state reconstruction of the patella in Mesozoic mammals, with alternative placement of *Eomaia* as a stem therian as found by O'Leary et al. (2013). Maximum likelihood values are shown at nodes. Our results using this topology suggest that the ossified patella evolved up to six times. Parsimony reconstruction similarly suggests between 4-6 instances of independent evolution. Compare with Fig. 5.

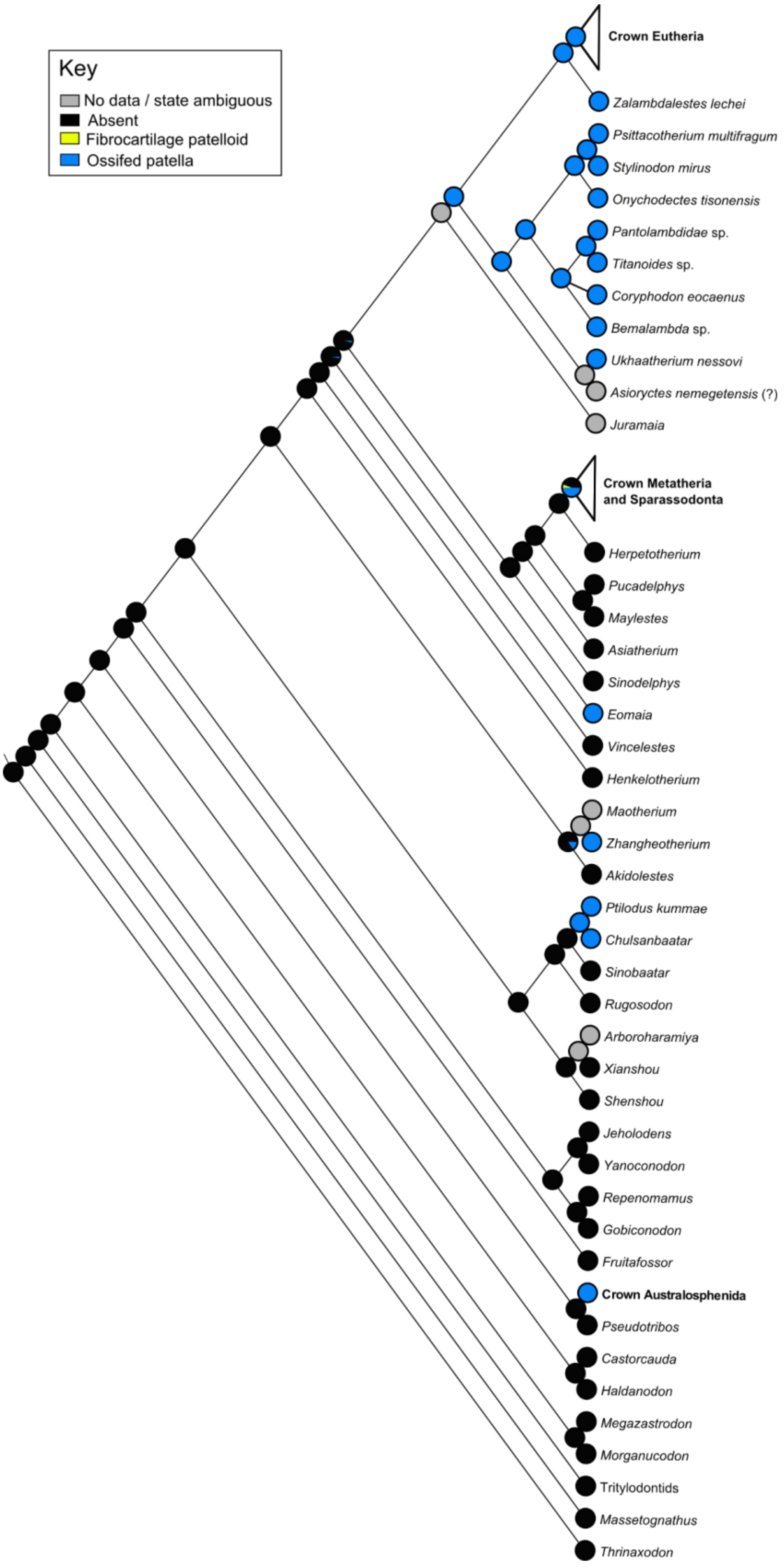

Supplementary Figure S5: Ancestral state reconstruction of the patella in Metatheria and related taxa, with alternative placement of Microbiotheriidae as shown by May-Collado et al. (2015). The results are similar to Fig. 6, suggesting one main instance of bony patellar evolution plus complex homoplasy afterwards, although likelihood values (shown at nodes) are slightly altered in favour of an ossified patella toward the base of Metatheria.

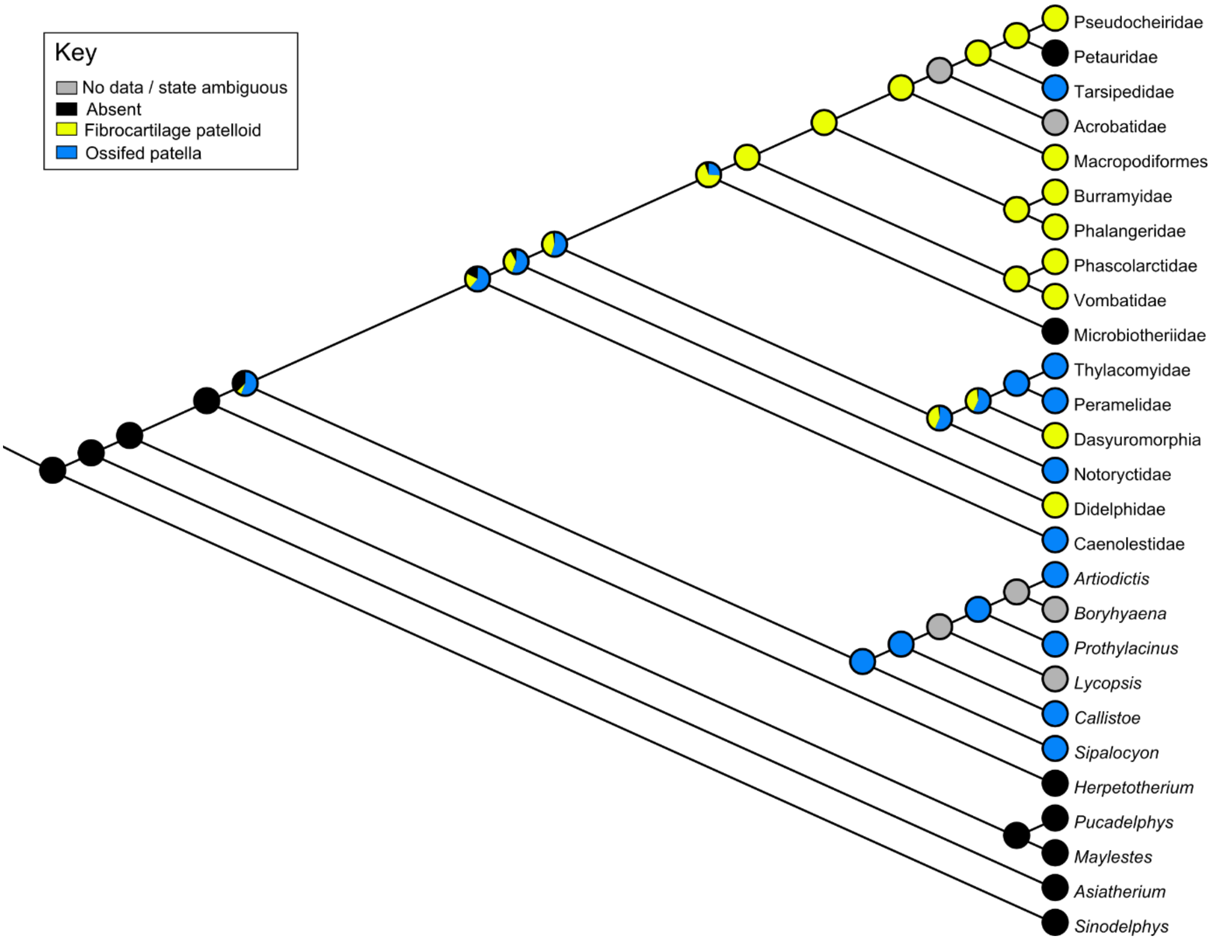

Supplementary References
(in addition to references provided to the main paper)

ABELLO M. A. & CANDELA, A. M. (2010). Postcranial skeleton of the Miocene marsupial *Palaeothentes* (Paucituberculata, Palaeothentidae): paleobiology and phylogeny. *Journal of Vertebrate Paleontology* **30,** 1515-1527.

ALEMSEGED Z., SPOOR, F., KIMBEL, W. H., BOBE, R., GERAADS, D., REED, D. & WYNN, J. G. (2006). A juvenile early hominin skeleton from Dikika, Ethiopia. *Nature* **443,** 296-301.

ANDREWS C. W. (1906). *A Descriptive Catalogue of the Tertiary Vertebrata of the Fayum, Egypt*. The British Museum of Natural History, London.

ANEMONE R. L. & COVERT, H. H. (2000). New skeletal remains of *Omomys* (Primates, Omomyidae): functional morphology of the hindlimb and locomotor behavior of a Middle Eocene primate. *J Hum Evol* **38,** 607-33.

ARGOT C. (2013). Postcranial analysis of a carnivoran-like archaic ungulate: The case of *Arctocyon primaevus* (Arctocyonidae, Mammalia) from the late Paleocene of France. *Journal of Mammalian Evolution* **20**, 83-114.

ARGOT C. & BABOT, J. (2011). Postcranial morphology, functional adapations and palaeobiology of *Callistoe vincei*, a predaceous metatherian from the Eocene of Salta, northwestern Argentina. *Palaeontology* **54,** 447-480.

BERTA A. (1987). The sabercat *Smilodon gracilis* from Florida and a discussion of its relationships (Mammalia, Felidae, Smilodontini). In *Bulletin of the Florida State Museum, Biological Sciences*, vol. 31, pp. 1-63. University of Florida, Gainesville.

BIN B. (2009). Osteology and sexual dimorphism of *Litolophus gobiensis*, and phylogenetic analysis of Eocene chalicotheres, Chinese Academy of Sciences.

BONAPARTE J. (2008). On the phylogenetic relationships of *Vincelestes neuquenianus*. *Historical Biology* **20,** 81-86.

BURNHAM D. A., ROTHSCHILD, B. M., BABIARZ, J. P. & MARTIN, L. D. (2013). Hemivertebrae as pathology and as a window to behavior in the fossil record. *Palarch's Journal of Vertebrate Palaeontology* **10,** 1-6.

CHOW M., ZHANG, Y., WANG, B. & DING, S. (1977). Paleocene mammalian fauna from the Nanxiong Basin, Guangdong Province. *Paleontologica Sinica* **153,** 1-88.

COOPER L. N., THEWISSEN, J. G. M., BAJPAI, S. & TIWARI, B. N. (2012). Postcranial morphology and locomotion of the Eocene raoellid *Indohys* (Artiodactyla: Mammalia). *HIstorical Biology* **24,** 279-310.

COPE E. D. (1873). On the flat clawed Carnivora of the Eocene of Wyoming, pp. 1-20. American Philosophical Society.

COPE E. D. (1883). The Vertebrata of the Tertiary formations of the West, vol. 1. U.S. Geological Survey, Dept. of the Interior, Washington, D.C.

COPE E. D. (1885). The Lemuroidea and the Insectivora of the Eocene period of North America. *American Naturalist* **19,** 457-471.

COPE E. D. & MATTHEW, W. D. (1915). Hitherto unpublished plates of Tertiary Mammalia and Permian Vertebrata, pp. 1-298. U.S. Geological Survey, Dept. of the Interior, Washington, D.C.

COURT N. (1994). Limb posture and gait in *Numidotherium koholense*, a primitive proboscidean from the Eocene of Algeria. *Zoological Journal of the Linnean Society* **111,** 297-338.

COUTO C. D. P. (1952). Fossil mammals from the beginning of the Cenozoic in Brazil. *Bulletin of the American Museum of Natural History* **99,** 355-394.

CZAPLEWSKI N. J., MORGAN, G. S. & MCLEOD, S. A. (2008). Chiroptera. In *Evolution of Tertiary Mammals of North America*, vol. 2 (ed. C. M. Janis, G. F. Gunnell and M. D. Uhen). Cambridge University Press, Cambridge.

DANAHER K., SHOCKEY, B. J. & MIHLBACHLER, M. (2009). Perissodactyl patellae: morphological variation and phylogenetic significance in a neglected element. In *Society of Vertebrate Paleontology Annual Conference*. Journal of Vertebrate Paleontology 28: 29 (suppl.): 85A.

DE MUIZON C. (1994). A new carnivorous marsupial from the Palaeocene of Bolivia and the problem of marsupial monophyly. *Nature* **370,** 208-211.

DE MUIZON C. (1998). *Mayulestes ferox*, a borhyaenoid (Metatheria, Mammalia) from the early Palaeocene of Bolivia. phylogenetic and paleobiologic implications. *Geodiversitas* **20,** 19-142.

DE VRIESE B. (1909). Recherches sur l' anatomie comparée de la rotule. *Bulletin of the Royal Academy of Medicine of Belgium*.

DELMER C., MAHBOUBI, M., TABUCE, R. & TASSY, P. (2006). A new species of *Moeritherium* (Proboscidea, Mammalia) from the Eocene of Algeria: new perspectives on the ancestral morphotype of the genus. *Palaeontology* **49,** 421-434.

DOMNING D. P. & BARNES, L. G. (2007). A new name for the 'Stanford skeleton' of *Paleoparadoxia* (Mammalia, Desmostylia). *Journal of Vertebrate Paleontology* **27,** 748-751.

DUNN J. F. & RASMUSSEN, T. (2007). Skeletal morphology and locomotor behavior of *Pseudotomus eugenei* (Rodentia, Paramyinae) from the Uinta Formation, Utah. *Journal of Vertebrate Paleontology* **27,** 987-1006.

DUNN R. H. (2009). Mammalian postcranial evolution and primate extinction in the middle Eocene of North America, Washington University.

ERFURT J. & METAIS, G. (2007). Endemic European Paleogene Artiodactyls. In *The Evolution of Artiodactyls* (ed. D. R. Prothero and S. E. Foss). Johns Hopkins Press, Baltimore.

ERICKSON B. (1962). A description of *Castoroides ohioensis* from Minnesota. P*roceedings of the Minnesota Academy of Science* **30**, 6–13.

FORASIEPI A. M. (2004). Anatomy of *Artiodictis sinclairi* (Mammalia, Metatheria) and the phylogenetic relationships of Sparassodonta, large carnivores of the Cenozoic of South PhD Thesis, University of Louisville.

FRANZEN J. L. (1992). The Messel horse show and other odd-toed ungulates. In *Messel: An insight into the history of life and of the earth* (ed. S. Schaal and W. Ziegler). Clarendon Press, London.

FRANZEN J. L., GINGERICH, P. D., HABERSETZER, J., HURUM, J. H., VON KOENIGSWALD, W. & SMITH, B. H. (2009). Complete primate skeleton from the Middle Eocene of Messel in Germany: morphology and paleobiology. *PLoS One* **4,** e5723.

GALBREATH E. C. (1955). Skull and skeleton of a mustelid, *Brachypsalis*, from the Miocene of northeastern Colorado. *University of Kansas Paleontological Contributions, Vertebrata* **20,** 1-15.

GAZIN C. L. (1936). A study of the fossil horse remains from the Upper Pliocene of Idaho. *Proceedings of the United States National Museum* **83,** 281-320.

GEBO D. L., SMITH, T. & DAGOSTO, M. (2012). New postcranial elements for the earliest Eocene fossil primate *Teilhardina belgica*. *J Hum Evol* **63,** 205-18.

GILMORE C. W. (1906). Notes on a newly mounted skeleton of *Merycoidodon*, a fossil mammal. *Proceedings of the United States National Museum* **31,** 513-514.

GINGERICH P. D. & DEUTCH, H. A. (1989). Systematics and evolution of early Eocene Hyaenodontidae (Mammalia, Creodonta) in the Clarks Fork Basin, Wyoming. *Contributions from the Museum of Anthropology University of Michigan* **27,** 327-391.

GINGERICH P. D., UL-HAQ, M., VON KOENIGSWALD, W., SANDERS, W. J., SMITH, B. H. & ZALMOUT, I. S. (2009). New protocetid whale from the middle eocene of Pakistan: birth on land, precocial development, and sexual dimorphism. *PLoS One* **4,** e4366.

GRANGER W. & SIMPSON, G. G. (1929). A revision of the Tertiary Multituberculata. *Bulletin of the American Museum of Natural History* **56,** 601-676.

GUNNELL G. F. (2008). Dinocerata. In *Evolution of Tertiary Mammals of North America*, vol. 1 (ed. C. M. Janis, G. F. Gunnell and M. D. Uhen). Cambridge University Press, Cambridge.

GUNNELL G. F. & GINGERICH, P. D. (1993). Skeleton of *Brachianodon westorum*, a new middle Eocene Metcheiromyid (Mammalia, Palaeanodonta) from the early Bridgerian (Bridger A) of the southern Green River Basin, Wyoming. *Contributions from the Museum of Paleontology University of Michigan* **28,** 365-392.

GUZIN C. L. (1965). A study of the early Tertiary condylarthran mammal *Meniscotherium*. *Smithsonian Miscellaneous Collection* **149,** 1-96.

HILL F. C. (1886). On the mounting of fossils. In *The American Naturalist* (ed. A. S. Packard and E. D. Cope), pp. 353-359. Essex Institute, Philadelphia.

HOROVITZ I., LADEVEZE, S., ARGOT, C., MACRINI, T., MARTIN, T., HOOKER, J. J., KURZ, C., DE MUIZON, C. & SANCHEZ-VILLAGRA, M. (2008). The anatomy of *Herpetotherium* cf. *fugax* CoPE, 1873, a metatherian from the Oligocene of North America. *Palaeontographica Abteilung A* **284,** 109-141.

HOROVITZ I., MARTIN, T., BLOCH, J., LADEVEZE, S., KURZ, C. & SANCHEZ-VILLAGRA, M. R. (2009). Cranial anatomy of the earliest marsupials and the origin of opossums. *PLoS One* **4,** e8278.

HU Y., MENG, J., WANG, Y. & LI, C. (2005). Large Mesozoic mammals fed on young dinosaurs. *Nature* **433,** 149-52.

HU Y., WANG, Y., LUO, Z. & LI, C. (1997). A new symmetrodont mammal from China and its implications for mammalian evolution. *Nature* **390,** 137-42.

INUZUKA N. (2009). The skeleton of *Desmostylus* from Utanobori, Hokkaido, Japan, II. Postcranial skeleton. *Bulletin of the Geological Survey of Japan* **60,** 257-379.

JENKINS F. A., JR., GATESY, S. M., SHUBIN, N. H. & AMARAL, W. W. (1997). Haramiyids and Triassic mammalian evolution. *Nature* **385,** 715-8.

JENKINS F. A., JR. & PARRINGTON, F. R. (1976). The postcranial skeletons of the Triassic mammals *Eozostrodon*, *Megazostrodon* and *Erythrotherium*. *Philos Trans R Soc Lond B Biol Sci* **273,** 387-431.

JENKINS F. A., JR. & SCHAFF, C. R. (1988). The early Cretaceous mammal *Gobiconodon* (Mammalia, Triconodonta) from the Cloverly formation in Montana. *Journal of Vertebrate Paleontology* **8,** 1-24.

JENKINS F. A. & KRAUSE, B. L. (1983). The postcranial skeleton of North American multituberculates. *Bulletin of the Museum of Comparative Zoology of Harvard University* **150,** 199-246.

JI Q., LUO, Z. X. & JI, S. A. (1999). A Chinese triconodont mammal and mosaic evolution of the mammalian skeleton. *Nature* **398,** 326-30.

JI Q., LUO, Z. X., YUAN, C. X. & TABRUM, A. R. (2006). A swimming mammaliaform from the Middle Jurassic and ecomorphological diversification of early mammals. *Science* **311,** 1123-7.

JI Q., LUO, Z. X., ZHANG, X., YUAN, C. X. & XU, L. (2009). Evolutionary development of the middle ear in Mesozoic therian mammals. *Science* **326,** 278-81.

JOHNSON K. A. & WALTON, D. W. (1989). Notoryctidae. In *Fauna of Australia*, vol. 1B (ed. D. W. Walton and B. J. Richardson). CSIRO Publishing.

KIELAN-JAWOROWSKA Z. & GAMBARYAN, P. P. (1994). Postcranial anatomy and habits of Asian multituberculate mammals. *Fossils and Strata* **36,** 3-92.

KOENIGSWALD W., ROSE, K. D., GRANDE, L. & MARTIN, R. D. (2005). First apatemyid skeleton from the Lower Eocene Fossil Butte Member, Wyoming (USA), compared to the European apatemyid from Messel, Germany. *Palaeontographica Abteilung A* **272,** 149-169.

KONDRASHOV P. & AGADJANIAN, A. K. (2012). A nearly complete skeleton of *Ernanodon* (Mammalia,Palaeanodonta) from Mongolia: morphofunctional analysis. *Journal of Vertebrate Paleontology* **32,** 983-1001.

KONDRASHOV P. & LUCAS, S. G. (2012). Nearly complete skeleton of *Tetraclaenodon* (Mammalia, Phenacodontidae) from the Early Paleocene of New Mexico: Morpho-functional analysis. *Journal of Paleontology* **86,** 25-43.

LEIDY J. (1869). The extinct mammalian fauna of Dakota and Nebraska. In *Journal of the Academy of Natural Sciences of Philadelphia, second series*, vol. 7, pp. 537. Academy of Natural Sciences, Philadelpha.

LEIDY J. (1873). Contributions to the extinct vertebrate fauna of the western territories. In *Report of the US Geological Survey of the Territories*, vol. 1 (ed. F. V. Hayden). Department of the Interior, Washington, D.C.

LORENTE M., GELFO, J. N. & LOPEZ, G. M. (2014). Postcranial anatomy of the early notoungulate *Allalmeia atalaensis* from the Eocene of Argentina. *Alcheringa: An Australasian Journal of Palaeontology* **38,** 1-14.

LUDTKE J. A. (2007). Family Agriochoeridae. In *The Evolution of Artiodactyls* (ed. D. R. Prothero and S. E. Foss). Johns Hopkins Press, Baltimore.

LULL R. S. (1905). *Megacerops Tyleri*, a New Species of Titanothere from the Bad Lands of South Dakota. *The Journal of Geology* **13,** 443-456.

LUO Z. X., CHEN, P., LI, G. & CHEN, M. (2007). A new eutriconodont mammal and evolutionary development in early mammals. *Nature* **446,** 288-93.

MACRINI T., DE MUIZON, C., CIFELLI, R. & ROWE, T. (2007). Digital cranial endocast of *Pucadelphys andinus*, a Paleocene metatherian. *Journal of Vertebrate Paleontology* **27,** 99-107.

MARTIN T. (2005a). Mandible and dentition of *Asfaltomylos patagonicus* (Australosphenida, Mammalia) and the evolution of tribosphenic teeth. *Journal of Vertebrate Paleontology* **25,** 414-425.

MARTIN T. (2005b). Postcranial anatomy of *Haldanodon exspectatus* (Mammalia, Docodonta) from the Late Jurassic (Kimmeridgian) of Portugal and its bearing for mammalian evolution. *Zoological Journal of the Linnean Society* **145,** 219-248.

MATTHEW W. D. (1897). A revision of the Puerco fauna. *Bulletin of the American Museum of Natural History* **19,** 259-323.

MATTHEW W. D. (1910). On the osteology and relationships of *Paramys*, and the affinities of the Ischryomyidae. *Bulletin of the American Museum of Natural History* **28,** 43-73.

MATTHEW W. D. (1937). Paleocene faunas of the San Juan basin, New Mexico. *Transactions of the American Philosophical Society* **30,** 1-510.

MAY-COLLADO L. J., KILPATRICK, C. W. & AGNARSSON, I. (2015). Mammals from 'down under': a multi-gene species-level phylogeny of marsupial mammals (Mammalia, Metatheria). *PeerJ* **3,** e805.

MCLAUGHLIN B., MIHLBACHLER, M. & ELLISON, M. (2010). The postcranial skeleton of *Embolotherium* (Brontotheriidae) from the middle and late Eocene of central Asia. In *Society of Vertebrate Paleontology Annual Conference*. Society of Vertebrate Paleontology, Pittsburgh.

MENG J., HU, Y. & LI, C. (2003). The osteology of *Rhombomylus* (Mammalia, Glires): Implications for phylogeny and evolution of Glires. *Bulletin of the American Museum of Natural History* **275,** 1-247.

MENG J., HU, Y., WANG, Y., WANG, X. & LI, C. (2006). A Mesozoic gliding mammal from northeastern China. *Nature* **444,** 889-93.

MENG J., WANG, Y. & LI, C. (2011). Transitional mammalian middle ear from a new Cretaceous Jehol eutriconodont. *Nature* **472,** 181-5.

MEREDITH R. W., KRAJEWSKI, C., WESTERMAN, M. & SPRINGER, M. S. (2009). Relationships and divergence times among the orders and families of Marsupialia. *Museum of Northern Arizona Bulletin* **65,** 383-406.

MIHLBACHLER M. C., LUCAS, S. G., EMRY, R. J. & BAYSHASHOV, B. (2004). A new brontothere (Brontotheriidae, Perissodactyla, Mammalia) from the Eocene of the Ily Basin of Kazakstan and a phylogeny of Asian ''horned'' brontotheres. *American Museum Novitates* **3439,** 1-42.

MIKKELSEN T. S., WAKEFIELD, M. J., AKEN, B., AMEMIYA, C. T., CHANG, J. L., DUKE, S., GARBER, M., GENTLES, A. J., GOODSTADT, L., HEGER, A., JURKA, J., KAMAL, M., MAUCELI, E., SEARLE, S. M., SHARPE, T., BAKER, M. L., BATZER, M. A., BENOS, P. V., BELOV, K., CLAMP, M., COOK, A., CUFF, J., DAS, R., DAVIDOW, L., DEAKIN, J. E., FAZZARI, M. J., GLASS, J. L., GRABHERR, M., GREALLY, J. M., GU, W., HORE, T. A., HUTTLEY, G. A., KLEBER, M., JIRTLE, R. L., KOINA, E., LEE, J. T., MAHONY, S., MARRA, M. A., MILLER, R. D., NICHOLLS, R. D., ODA, M., PAPENFUSS, A. T., PARRA, Z. E., POLLOCK, D. D., RAY, D. A., SCHEIN, J. E., SPEED, T. P., THOMPSON, K., VANDEBERG, J. L., WADE, C. M., WALKER, J. A., WATERS, P. D., WEBBER, C., WEIDMAN, J. R., XIE, X., ZODY, M. C., GRAVES, J. A., PONTING, C. P., BREEN, M., SAMOLLOW, P. B., LANDER, E. S. & LINDBLAD-TOH, K. (2007). Genome of the marsupial *Monodelphis domestica* reveals innovation in non-coding sequences. *Nature* **447,** 167-77.

MITCHELL K. J., PRATT, R. C., WATSON, L. N., GIBB, G. C., LLAMAS, B., KASPER, M., EDSON, J., HOPWOOD, B., MALE, D., ARMSTRONG, K., MEYER, M., HOFREITER, M., AUSTIN, J., DONNELLAN, S. C., LEE, M. S., PHILLIPS, M. J. & COOPER, A. (2014). Molecular phylogeny, biogeography, and habitat preference evolution of marsupials. *Molecular Biology and Evolution*, msu176.

MOLINERO R. (2003). Comparative anatomy of *Henkelotherium guimarotae* (Holotheria), a Late Jurassic small mammal, and its relevance for the evolution of the mode of locomotion of modern mammals. PhD thesis, Freie Universität Berlin.

MORGAN G. S., LANDER, E. B., CIKOSKI, C., CHAMBELIN, R. M., LOVE, D. W. & PETERS, L. (2009). The oreodont *Merychyus major major* (Mammalia: Artiodactyla: Oreodontidae) from the Miocene Popotosa Formation, Bosque del Apache National Wildlife Refuge, Socorro County, central New Mexico. *New Mexico Geology* **31,** 91-103.

NAKATSUKASA M., KUNIMATSU, Y., SHIMIZU, D., NAKANA, Y., KIKUCHI, Y. & ISHIDA, H. (2012). Hind limb of the *Nacholapithecus kerioi* holotype and implications for its positional behavior. *Anthropological Science* **120,** 235-250.

O'LEARY M. A. & ROSE, K. D. (1995). Postcranial skeleton of the early Eocene mesonychid *Pachyaena* (Mammalia: Mesonychia). *Journal of Vertebrate Paleontology* **15,** 401-430.

OSBORN H. F. (1898). Remounted skeleton of *Phenacodus primaevus*. Comparison with Euprotogonia. *Bulletin of the American Museum of Natural History* **10,** 159-164.

OSBORN H. F. (1900). *Oxyaena* and *Patriofelis* restudied as terrestrial creodonts. *Bulletin of the American Museum of Natural History* **13,** 269-279.

OSGOOD W. H. (1921). A monographic study of the American marsupial, *Caenolestes*, vol. 14, pp. 1-162. Field Museum of Natural History, Chicago.

PATTERSON B. (1934). A contribution to the osteology of *Titanoides* and the relationships of the Amblypoda. *Transactions of the American Philosophical Society* **73,** 71-101.

PETERSON O. A. (1909). A revision of the Enteledontidae. In *Memoirs of the Carnegie Museum*, vol. 4, pp. 156.

PICKFORD M., SENUT, B., MUSALIZI, S. & MUSIIME, E. (2013). The osteology of *Nonanomalurus soniae*, a non-volant arboreal rodent (Mammalia) from the Early Miocene of Napak, Uganda. *Geo-Pal Uganda* **7,** 1-33.

ROSE K. D. (1985). Comparative osteology of North American dichobunid artiodactyls. *Journal of Paleontology* **59,** 1202-1226.

ROSE K. D. (1987). Climbing adaptations in the early eocene mammal *Chriacus* and the origin of Artiodactyla. *Science* **236,** 314-6.

ROSE K. D., CHEW, A. E., DUNN, R. H., KRAUS, M. J., FRICKE, H. C. & ZACK, S. P. (2012). Earliest Eocene mammalian fauna from the paleocene-eocene thermal maximum at Sand Creek Divid, Souther Bighorn Basin, Wyoming. In *Papers in Paleontology*, vol. 36. University of Michigan.

ROUGIER G. W. (1993). *Vincelestes neuquenianus* Bonaparte (Mammalia,Theria) un primitivo mamı´fero del Creta´cico Inferior de la Cuenca Neuquina. PhD thesis, University of Buenos Aires, Argentina.

ROUGIER G. W., WIBLE, J. R. & NOVACEK, M. J. (1998). Implications of *Deltatheridium* specimens for early marsupial history. *Nature* **396,** 459-63.

SCHLOSSER M. (1887). Die Affen, Lemuren, Chiropteren, Insectivoren, Marsupialen, Creodonten und Carnivoren des europaischen Tertiars und deren Beziehungen zu ihren lebenden und fossilen aussereuropaischen Verwandten. In *Beitrage zur Palaontologie osterreich-ungarns und des orients*, vol. 6 (ed. E. v. Mojsisovics and M. Neumayr), pp. 1-162. Holder, Wien.

SCHOCH R. M. (1986). Systematics, functional morphology and macroevolution of the extinct mammalian order Taeniodonta. *Bulletin of the Peabody Museum of Natural History* **42,** 1-307.

SCHOCKEY B. J. (2001). Specialized knee joints in some extinct, endemic, South American herbivores. *Acta Palaeontologica Polonica* **46,** 277-288.

SCHULTZ A. H. (1960). Einige Beobachtungen und Maße am Skelett von Oreopithecus: im Vergleich mit anderen catarrhinen Primaten. *Zeitschrift für Morphologie und Anthropologie* **50,** 136-149.

SCOTT W. B., JEPSEN, G. L. & ELMER, A. (1937). The Mammalian Fauna of the White River Oligocene: Part II. Rodentia. *Transactions of the American Philosophical Society, New Series* **28,** 155-269.

SHOCKEY B. J. (2001). Specialized knee joints in some extinct, endemic, South American herbivores. *Acta Palaeontologica Polonica* **46,** 277-288.

SIMONS E. (1960). The Paleocene Pantodonta. *Transactions of the American Philosophical Society new series* **50,** 3-99.

SIMPSON G. G. & ELFTMAN, H. O. (1928). Hind limb musculature and habits of a paleocene multituberculate. *American Museum Novitates* **333,** 1-19.

SINCLAIR W. J. (1905). The marsupial fauna of the Santa Cruz beds. *Proceedings of the American Philosophical Society* **44,** 73-81.

SINCLAIR W. J. (1925). The mounted skeleton of a new *Mesohippus* from the Protoceras beds. *Proceedings of the American Philosophical Society* **64,** 55-63.

SPAULDING M. & FLYNN, J. J. (2012). Phylogeny of the Carnivoramorpha: The impact of postcranial characters. *Journal of Systematic Paleontology* **10,** 63-677.

SPRINGHORN R. (1992). Carnivores: agile climbers and prey catchers. In *Messel: An insight into the history of life and of the earth* (ed. S. Schaal and W. Ziegler). Clarendon Press, London.

STORCH G. & RICHTER, G. (1992). Pangolins: almost unchanged for 50 million years. In *Messel: An insight into the history of life and of the earth* (ed. S. Schaal and W. Ziegler). Clarendon Press, London.

STORCH G., SIGE, B. & HABERSETZER, J. (2002). *Tachypteron franzeni* n. gen., n. sp., earliest emballonurid bat from the Middle Eocene of Messel (Mammalia, Chiroptera) *Palaiontologische Zeitschrift* **76,** 189-199.

SULLIVAN J. A. (2003). A new species of *Archaeohippus* (Mammalia, Equidae) from the Arikareean of central Florida. *Journal of Vertebrate Paleontology* **23,** 877-885.

SUSMAN R. (1988). New postcranial remains from Swartkrans and their bearing on the functional morphology and behavior of *Paranthropus robustus*. In *Evolutionary History of the "Robust" Australopithecines* (ed. F. E. Grine), pp. 149-162. Transaction Publishers, New Jersey.

TRINKHAUS E. (2000). Human patellar articular proportions: recent and Pleistocene patterns. *Journal of Anatomy* **196,** 473-483.

TROFIMOV B. A. & SZALAY, F. S. (1994). New Cretaceous marsupial from Mongolia and the early radiation of Metatheria. *Proc Natl Acad Sci U S A* **91,** 12569-73.

TURNER A. H. & ANTON, M. (1997). *The Big Cats and their Fossil Relatives*. Columbia University Press, New York.

VAZQUEZ-MOLINERO R., MARTIN, T., FISCHER, M. S. & FREY, R. (2001). Comparative anatomical investigations of the postcranial skeleton of *Henkelotherium guimarotae* Krebs, 1991 (Eupantotheria, Mammalia) and their implications for its locomotion. *Mitt. Mus. Nat.kd. Berl., Zoo!. Reihe* **77,** 207-216.

VON KOENIGSWALD W., STORCH, G. & RICHTER. (G.1992). Primitive insectivores, extraordinary hedgehogs and long-fingers. In *Messel: An insight into the history of life and of the earth* (ed. S. Schaal and W. Ziegler). Clarendon Press, London.

WARBURTON N. M. (2004). Functional morphology and evolution of marsupial moles (Marsupialia; Notoryctomorphia), University of Western Australia.

WARBURTON N. M. (2006). Functional morphology of marsupial moles (Marsupialia: Notoryctidae). *Verhandlungen des Naturwissenschaftlichen Vereins in Hamburg* **42,** 39-149.

WARBURTON N. M., WOOD, C., LLOYD, C., SONG, S. & WITHERS, P. C. (2003). The 3-dimensional anatomy of the north-western marsupial mole (*Notoryctes caurinus*, Thomas 1920) using computed tomography, X-ray and magnetic resonance imaging. *Records of the Western Australian Museum* **22,** 1-7.

WARD C. V., RUFF, C. B., WALKER, A., TEAFORD, M. F., ROSE, M. D. & NENGO, I. O. (1995). Functional morphology of *Proconsul* patellas from Rusinga Island, Kenya, with implications for other Miocene-Pliocene catarrhines. *J Hum Evol* **29,** 1-19.

WATERHOUSE G. R. (1846). Marsupiata, or pouched animals. In *A Natural History of the Mammalia*, vol. 1. Hippolyte Bailliere, London.

WHEELER W. H. (1961). Revision of the Uintatheres. In *Peabody Museum of Natural History*, vol. 14. Yale University, New Haven.

WILLIAMSON T. E., BRUSATTE, S. L., CARR, T. D., WEIL, A. & STANDHARDT, B. R. (2012). The phylogeny and evolution of Cretaceous–Palaeogene metatherians: cladistic analysis and description of new early Palaeocene specimens from the Nacimiento Formation, New Mexico. *Journal of Systematic Palaeontology* **10,** 625-651.

WOOD A. E. (1962). The early Tertiary rodents of the family Paramyidae. *Transactions of the American Philosophical Society, New Series* **51,** 3-261.

WOOD A. R., BEBEJ, R. M., MANZ, C. L., BEGUN, D. L. & GINGERICH, P. D. (2011). Postcranial functional morphology of *Hyracotherium* (Equidae, Perissodactyla) and locomotion in the earliest horses. *Journal of Mammalian Evolution* **18,** 1-32.

WOODMAN N. & BRANSTRATOR, J. W. (2008). The Overmyer Mastodon (*Mammut americanum*) from Fulton County, Indiana. *The American Midland Naturalist* **159,** 125-146.

WORTMAN J. L. (1894). Osteology of *Patriofelis*, a middle Eocene Creodont. *Bulletin of the American Museum of Natural History* **6,** 129-165.

YANOFSKY A. (1998). Stanford *Paleoparadoxia* fossil skeleton mounting, pp. 1-143. Stanford Linear Accelerator Publications, Stanford.

ZAPFE H. (1958). The skeleton of *Pliopithecus* (*Epipliopithecus*) *vindobonensis* Zapfe and Hürzeler. *American Journal of Physical Anthropology* **16,** 441-457.

ZHOU X., SANDERS, W. J. & GINGERICH, P. D. (1992). Functional and behavioral implications of vertebral structure in *Pachyaena ossifraga* (Mammalia, Mesonychia). *Contributions from the Museum of Paleontology University of Michigan* **28,** 289-319.
